# Long-lasting forms of plasticity through patterned ultrasound-induced brainwave entrainment

**DOI:** 10.1126/sciadv.adk3198

**Published:** 2024-02-23

**Authors:** Ho-Jeong Kim, Tien Thuy Phan, Keunhyung Lee, Jeong Sook Kim, Sang-Yeong Lee, Jung Moo Lee, Jongrok Do, Doyun Lee, Sung-Phil Kim, Kyu Pil Lee, Jinhyoung Park, C. Justin Lee, Joo Min Park

**Affiliations:** ^1^ Center for Cognition and Sociality, Institute for Basic Science, Daejeon, Republic of Korea.; ^2^ Department of Biomedical Engineering, Ulsan National Institute of Science and Technology (UNIST), Ulsan, Republic of Korea.; ^3^ University of Science and Technology (UST), Daejeon, Republic of Korea.; ^4^ Department of Intelligent Precision Healthcare Convergence, Sungkyunkwan University, Suwon, Republic of Korea.; ^5^ Department of Biomedical Engineering, Sungkyunkwan University, Suwon, Republic of Korea.; ^6^ Department of Physiology, College of Veterinary Medicine, Chungnam National University, Daejeon, Republic of Korea.; ^7^ Department of Biological Sciences, Korea Advanced Institute of Science and Technology, Daejeon 34141, Republic of Korea.; ^8^ Department of Biomedical Engineering, Sungkyunkwan University, Suwon, Republic of Korea.

## Abstract

Achieving long-lasting neuronal modulation with low-intensity, low-frequency ultrasound is challenging. Here, we devised theta burst ultrasound stimulation (TBUS) with gamma bursts for brain entrainment and modulation of neuronal plasticity in the mouse motor cortex. We demonstrate that two types of TBUS, intermittent and continuous TBUS, induce bidirectional long-term potentiation or depression-like plasticity, respectively, as evidenced by changes in motor-evoked potentials. These effects depended on molecular pathways associated with long-term plasticity, including *N*-methyl-d-aspartate receptor and brain-derived neurotrophic factor/tropomyosin receptor kinase B activation, as well as de novo protein synthesis. Notably, bestrophin-1 and transient receptor potential ankyrin 1 play important roles in these enduring effects. Moreover, pretraining TBUS enhances the acquisition of previously unidentified motor skills. Our study unveils a promising protocol for ultrasound neuromodulation, enabling noninvasive and sustained modulation of brain function.

## Teaser

Patterned ultrasound stimulation bidirectionally modulates long-term neuroplasticity.

## INTRODUCTION

Noninvasive brain stimulation (NIBS) techniques, such as transcranial magnetic stimulation (TMS) and transcranial direct and alternating current stimulation, have gained considerable attention for their potential to modulate brain function in humans (*1*, *2*). However, these magnetic and electrical NIBS methods have inherent limitations that restrict their spatial resolution and penetration depth, making it challenging to precisely stimulate specific brain regions with optimal efficacy (*3*, *4*). Therefore, there is a pressing need to develop alternative approaches that can overcome these limitations and provide more efficient and precise modulation of brain function.

In recent years, low-intensity, low-frequency ultrasound stimulation (LILFUS) has emerged as a promising technique with the potential to address the limitations of traditional NIBS methods. Unlike magnetic or electrical stimulation, LILFUS can be introduced deep into brain tissue (several millimeters to more than several tens of millimeters at 500 to 650 kHz) with high spatial resolution ranging from less than one to several cubic millimeters and produce local changes in brain physiology (*5*, *6*). This unique capability offers exciting prospects for precise and focused modulation of specific brain regions, allowing for a more refined understanding of their functional contributions.

Previous studies have indicated that transcranial ultrasound stimulation can affect synaptic plasticity, such as long-term potentiation (LTP) or depression (LTD). However, the neuromodulatory effects of transcranial ultrasound stimulation are generally weak and rarely last longer than 10 min (*7*, *8*). Although recent research on anesthetized rats has shown sustained depression in hippocampal synaptic connectivity following transcranial focused ultrasound stimulation (tFUS) (*9*), the long-term changes induced by ultrasound in brain function, their underlying mechanisms, and their connection to learning and memory processes are not yet fully understood.

In this study, we have established specific ultrasound parameters designed to mimic the brainwave patterns of theta and gamma oscillations observed during learning and memory processes (*10*–*12*). By entraining brain activity and replicating the oscillatory patterns associated with cognitive processes, we were able to induce predictable and long-lasting changes in brain function. These effects on brain functions rely on several well-known molecular and cellular markers that modulate brain plasticity. The precise modulation of specific brain regions and the induction of enduring changes in neural function through ultrasound stimulation hold great promise for innovative interventions in normal brain functions and various brain diseases.

## RESULT

### Modulation of motor-evoked potentials and long-lasting effects in motor cortex (M1) through theta burst ultrasound stimulation

**Fig. 1.**
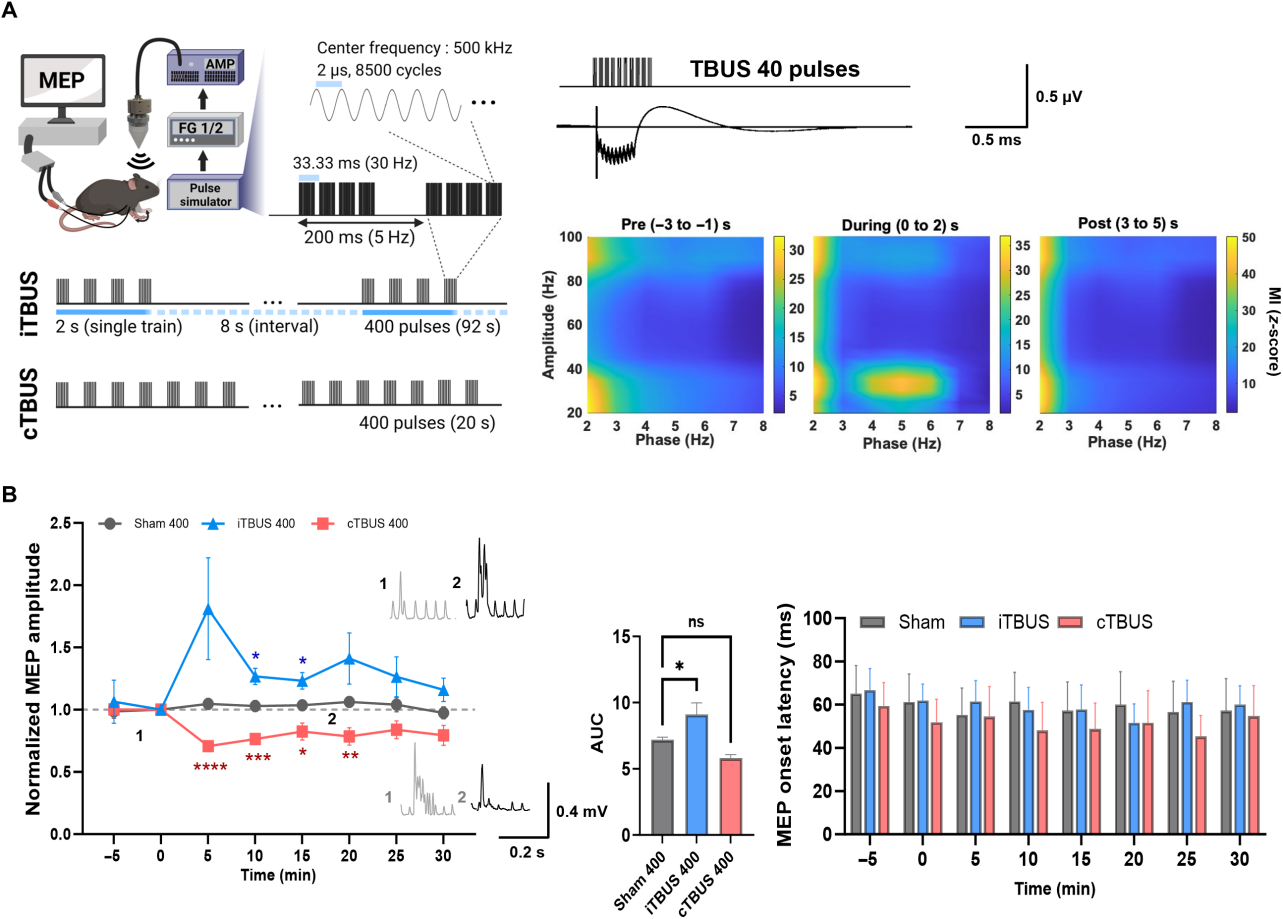
Bidirectional modulation of MEP responses and local entrainment of brain oscillations through transcranial ultrasound stimulation. (**A**) Experimental setup: 500-kHz ultrasound pulses with a duration of 17 ms were delivered in a theta burst pattern (four pulses at 30-Hz gamma frequency, repeated at 5-Hz theta frequency). Intermittent TBUS (iTBUS) involved 2-s theta burst trains repeated every 10 s, while continuous TBUS (cTBUS) consisted of uninterrupted repetitions of the same train. Subdermal electroencephalogram recordings demonstrated strong gamma-theta coupling during TBUS. *n* = 6 for each group. (**B**) MEP amplitude and onset latency were analyzed. The iTBUS group showed higher MEPs until 15 min, whereas the cTBUS group had lower MEPs from 5 to 15 min compared to the sham group. MEP onset latency did not considerably change. Error bands represent SEM. The inserted raw traces display representative traces at indicated time points. *n* = 6 for each group. Data were presented as means ± SEM. In the MEP trace, *P* values were determined by an unpaired two-tailed *t* test for each time point. In the area under the curve (AUC) of the MEP trace, the *P* value was determined by ordinary one-way analysis of variance (ANOVA) followed by Dunnett’s test. Every *P* value is *P < 0.05, **P < 0.01, ***P < 0.001, and ****P < 0.0001; ns, not significant.

To investigate the effects of ultrasound stimulation on brain function, we applied the noninvasive stimulation to the contralateral M1 of hotspot associated with the proximal forelimb muscle movement. Using the amplitude of motor-evoked potentials (MEPs) as a measure of cortical motor neuron activity, we were able to quantifiably assess the changes induced by the ultrasound stimulation (*13*, *14*). Our study demonstrated that individual pulses of ultrasound stimulus, characterized by a 0.5-MHz center frequency, 8500 continuous cycles over a 17-ms pulse duration, and a spatial-peak pulse-average intensity (*I*_sppa_) of 2.79-W/cm^2^, consistently elicited MEPs in the contralateral triceps muscle of mice (fig. S3A) (*15*). Note that these ultrasound parameters are comparatively weaker than those used in other studies involving cells, animals, and humans (*16*–*18*). On the basis of these findings, we developed a pioneering protocol called theta burst ultrasound stimulation (TBUS) coupled with gamma burst to modulate brain function and induce long-lasting effects bidirectionally. For the patterned ultrasound-induced brainwave entrainment, the TBUS protocol consisted of a 2-s theta-gamma coupling pattern, where ultrasound stimuli were delivered four times with a 33.33-ms interstimulus onset interval (gamma frequency of 30 Hz), repeated every 200 ms (at a theta frequency of 5 Hz) (Fig. 1A and fig. S3B). We used two variations of the protocol: intermittent TBUS (iTBUS) and continuous TBUS (cTBUS). For iTBUS, the 2-s train was delivered 10 times for 400 pulses, with an 8-s break interval between each train. For cTBUS, the protocol was performed continuously without break intervals, extending to 100 s for 400 pulses. We also investigated the effectiveness of the gamma frequency used in our protocol by varying the number of gamma pulses during TBUS. The results showed that using four gamma pulses per burst resulted in more remarkable benefits than using 3, 5, and 6 gamma pulses (fig. S4).

To assess the potential of TBUS in inducing phase-amplitude coupling (PAC) between theta and gamma rhythms involved in memory processing, we measured PAC using subdermal electroencephalogram (EEG) recordings. PAC was examined before, during, and after TBUS stimulation. Our findings revealed explicit synchronization between 5-Hz theta and 30-Hz gamma rhythms during TBUS stimulation, indicating the successful induction of PAC (Fig. 1A). However, we did not observe considerable after-effects in PAC compared to the resting state following TBUS stimulation. These results suggest that transcranial ultrasound stimulation, specifically theta-gamma coupling, has the ability to entrain ongoing oscillations in the brain locally.

### Bidirectional effects of patterned TBUS and the role of gamma burst

To evaluate the effects of TBUS, we compared the averaged amplitude of MEP in two MEP blocks (25 trials in each block) before TBUS. We then compared this baseline with the MEP blocks obtained after the TBUS application. TBUS was administered at 80% intensity, corresponding to subthreshold stimulation, using the single ultrasound stimulus for MEPs. The results demonstrated that iTBUS increased MEP amplitude (peak amplitude 181 ± 0.24% at 5 min; *P* = 0.004 versus baseline), while cTBUS led to a decrease in MEP amplitude (76 ± 0.24% at 10 min; *P* = 0.001 versus baseline) compared to the sham condition (Fig. 1B). The MEP onset latency was not immediate but had a delay of approximately 60 ms after the individual ultrasound pulse. However, MEP onset latency was not powerfully affected (Fig. 1B and fig. S3).

We experimented to compare the effects of iTBUS, using a pulse repetition frequency (PRF) of 10 Hz commonly used in LILFUS neuromodulation (*8*), on the LTP-like plasticity of MEP amplitude. The ultrasound intensity and pulse numbers (400 pulses) were consistent between the 10-Hz stimulation group and the iTBUS 400 group, with the only difference being the delivery pattern. The results revealed that iTBUS facilitated MEP amplitude evoked by a single ultrasound pulse (190 ± 0.28% at 10 min; *P* = 0.003 versus baseline), whereas the 10-Hz protocol applied after obtaining a stable baseline with a single ultrasound pulse had little effect on MEP amplitude (fig. S6A). These findings demonstrate that bidirectional effects on brain function can be achieved by adjusting the stimulation parameters and delivery patterns, highlighting the potential of ultrasound stimulation for finely modulating brain function to produce lasting effects.

Previous studies have specifically emphasized the functional importance of theta-gamma coupling rather than individual theta or gamma frequencies (*19*, *20*). To investigate the necessity of gamma bursts, we compared iTBUS with a modified stimulation protocol called i-Theta. i-Theta included the same number of pulses as iTBUS but only involved theta pattern stimulation without gamma bursts. Our results demonstrated that iTBUS had a notably superior effect on MEP amplitudes (167 ± 0.17% at 10 min; *P* = 0.003 versus baseline) compared to i-Theta stimulation (127 ± 0.14% at 5 min; *P* = 0.08 versus baseline) (fig. S6B). This finding is consistent with previous studies highlighting the functional importance of theta-gamma coupling in various cognitive tasks (*21*, *22*). It supports the notion that the effect of a nonlinear burst mode is more robust than that of a linear tonic mode with the same number of single spikes (*23*).

**Fig. 2.**
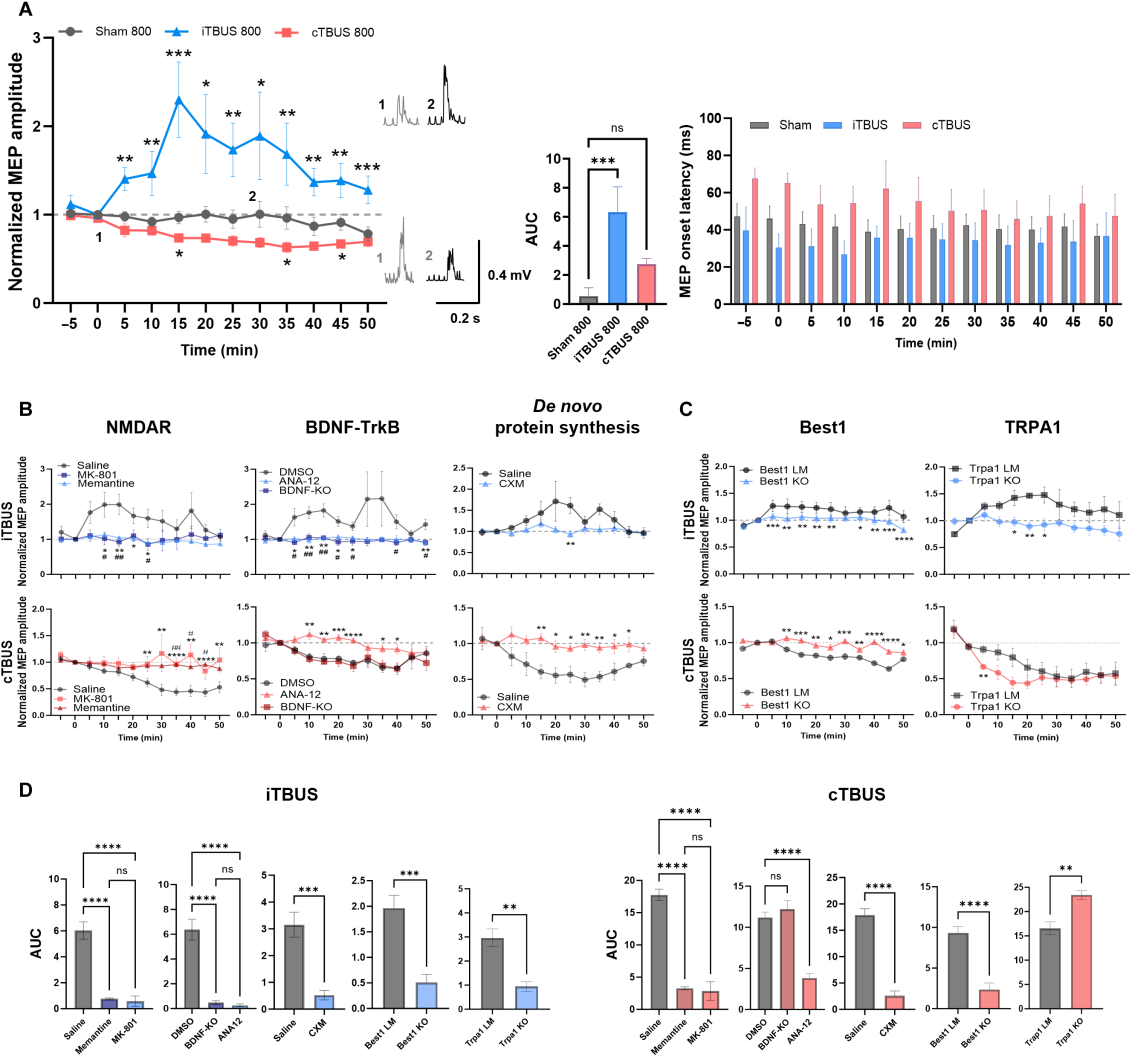
Longevity of TBUS effect and screening of molecular candidates in TBUS-induced MEP plasticity. (**A**) Optimization of stimulus parameters: 800 pulses. In the iTBUS group, MEP values were increased compared to baseline, while the cTBUS group showed decreased MEPs in block 8 (35 to 40 min). Data were presented as means ± SEM. In MEP trace, *P* values were determined by an unpaired two-tailed *t* test at each time point. In the AUC of the MEP trace, the *P* value was determined by ordinary one-way ANOVA followed by Dunnett’s test. (**B** and **C**) Group data of MEP amplitude for iTBUS (top) and cTBUS (bottom) with drug treatment, *n* = 6 for each group. Data were presented as means ± SEM. *P* values were determined by an unpaired two-tailed *t* test at each time point. (**D**) Conversion of MEP amplitude graph to an AUC graph. *P* values were determined by ordinary one-way ANOVA with Tukey’s test. The baseline of AUC = 1 and every *P* value is *P < 0.05, **P < 0.01, ***P < 0.001, and ****P < 0.0001; ns, not significant.

Next, we investigated the longevity of TBUS effects by increasing the number of pulses from 400 to 800. TBUS with 800 pulses induced long-lasting changes in MEP amplitude for both iTBUS (230 ± 0.31% at 15 min; *P* = 0.002 versus baseline) and cTBUS (52 ± 0.26% at 35 min; *P* = 0.024 versus baseline), with effects lasting for more than 50 min (Fig. 2A). These findings indicate that the duration of both iTBUS and cTBUS effects approximately doubled with 800 pulses. Through these experiments, we were able to elucidate the importance of gamma bursts in TBUS and demonstrate that increasing the number of pulses enhances the longevity of TBUS effects. In addition, we found that neither iTBUS nor cTBUS induced any substantial temperature alterations in the local brain region, affirming that the effects observed in MEP amplitude were not due to thermal changes (fig. S1C). Furthermore, we observed the induction of LTP- and LTD-like plasticity using stimulus conditions with a smoothed envelope (fig. S1C) (*24*–*26*).

### Involvement of NMDAR in ultrasound-induced plasticity in the motor cortex

*N*-Methyl-d-aspartate receptors (NMDARs) play a crucial role in various forms of bidirectional synaptic plasticity, such as LTP and LTD, throughout the neural pathway from the spinal cord to the cerebral cortex (*27*). In the human motor cortex, the acute activation of NMDAR is required to induce long-lasting effects on excitability by TMS (*28*). To investigate the potential involvement of NMDARs in the plasticity-like changes induced by ultrasound stimulation of the theta-gamma coupling pattern, we first tested whether TBUS effects were NMDAR-dependent. We conducted experiments using memantine (a partial NMDAR antagonist, 5 mg/kg, ip), low-dose d-cycloserine– (DCS; a partial NMDAR agonist, 3 mg/kg, ip), MK-801– (dizocilpine, an NMDAR antagonist, 0.1 mg/kg, ip), and saline-treated groups to determine whether the activation and inhibition of NMDARs are linked to the long-term effects of transcranial TBUS.

After applying iTBUS, the saline-treated group exhibited a powerful facilitation effect, indicated by increased MEP amplitude (152 ± 0.1% at 20 min; *P* = 0.0005 versus baseline). In contrast, the memantine- and MK-801–treated groups did not show important changes in MEP amplitude (Fig. 2B). The low-dose DCS-treated group initially displayed a brief attenuation of responses, followed by delayed facilitation starting from 30 min after iTBUS application (161 ± 1.1% at 50 min; *P* = 0.02 versus baseline) (fig. S7A). This aligns with previous observations of the effects of low-dose DCS in individuals undergoing intermittent theta burst TMS (*29*).

As expected, the saline-treated group exhibited an LTD-like effect with cTBUS (73 ± 0.06% at 25 min; *P* = 0.0002 versus baseline); however, the cTBUS effect was notably inhibited by the NMDAR blockers (Fig. 2B). The low-dose DCS-treated group initially exhibited slight facilitation effects but later displayed suppression effects following cTBUS (76 ± 0.5% at 50 min; *P* = 0.0003 versus baseline) with no impact on baseline level obtained by single ultrasound pulses (fig. S7A). This result is consistent with a previous report suggesting that the low-dose DCS can potentially be involved in LTP-like processes through augmented NMDAR signaling (*30*). Our findings indicate that NMDARs play a crucial role in the plasticity induced by TBUS in M1.

### BDNF/TrkB signaling and protein synthesis dependency in ultrasound-induced plasticity in the motor cortex

Brain-derived neurotrophic factor (BDNF) is critical for activity-dependent neuronal plasticity in the hippocampus and other brain regions and regulates protein synthesis (*31*–*33*). Therefore, we tested whether TBUS application is associated with BDNF and its receptor, tropomyosin receptor kinase B (TrkB). We treated subjects with ANA-12 (0.5 mg/kg, ip), an antagonist of TrkB receptors, and used a vehicle [0.5% of dimethyl sulfoxide (DMSO) in normal saline] as a control group.

Our results showed that the ANA-12–treated group did not exhibit critical TBUS-induced effects (Fig. 2B). In contrast, the DMSO group displayed meaningful LTP- and LTD-like plasticity of MEPs following TBUS (211 ± 0.32% at 35 min; *P* = 0.01 versus baseline for iTBUS; 65 ± 0.1% at 40 min; *P* = 0.003 versus baseline for cTBUS). These findings suggest the involvement of BDNF/TrkB signaling in mediating the observed plasticity effects.

To further investigate the contribution of activity-dependent BDNF, we used mice lacking activity-driven neuronal BDNF expression through promoter IV (BDNF-IV-KO) (*34*). BDNF is transcribed by multiple promoters, with promoter IV consequentially transcribing through activity-dependent regulation. We observed that LTP-like plasticity of MEP in BDNF-IV-KO mice was considerably impaired following iTBUS; however, LTD-like plasticity of MEP remained intact following cTBUS (Fig. 2B). These results suggest the possible requirement of promoter IV–dependent BDNF transcription as a mediator of LTP-like plasticity than LTD-like plasticity. This finding may be closely related to the role of BDNF in synaptic plasticity in the prefrontal cortex and its regulation of neural oscillation in behaving animals (*35*, *36*).

Since long-term synaptic plasticity requires new protein synthesis to facilitate changes in synaptic strength (*37*), we investigated the effect of cycloheximide (CXM; 30 mg/kg, ip), a protein synthesis inhibitor, on TBUS-induced plasticity. We observed that the CXM-treated group did not exhibit crucial MEP changes following iTBUS, whereas the saline-treated group displayed LTP-like effects (171 ± 0.25% at 20 min; *P* = 0.0001 versus baseline). Similarly, following cTBUS, the CXM-treated group did not show any notable changes, while the saline-treated group exhibited the LTD-like effect until the end of the recording (49 ± 0.1% at 30 min; *P* = 0.002 versus baseline). These results indicate that activity-dependent BDNF/TrkB signaling and de novo protein synthesis are required for long-lasting effects of both iTBUS and cTBUS.

### Involvement of TRPA1 and Best1 as gateways to TBUS-induced plasticity

Low-intensity ultrasonic stimulations using frequencies less than 1 MHz have modulated neuronal activity directly or indirectly via nonthermal, mechanical effects (*6*, *18*, *38*). Notably, low-intensity ultrasound stimulation activates astrocytic transient receptor potential ankyrin 1 (TRPA1). This ion channel perceives the LILFUS signal and participates in a potential mechanism for low-intensity sonogenetic neuromodulation in vitro and in vivo (*38*, *39*). To examine the role of TRPA1 in TBUS-induced plasticity, we used HC-030031, a selective TRPA1 inhibitor, and conducted MEP recordings in wild-type (WT) mice. We administered HC-030031 (20 or 100 mg/kg) and recorded MEPs in WT mice. HC-030031 strongly attenuated the plasticity of MEP amplitude induced by iTBUS compared to the vehicle-treated group. Moreover, HC-030031 partially suppressed the LTD-like plasticity of MEP following cTBUS (fig. S5B). These findings suggest that TRPA1 inhibition plays a role in modulating TBUS-induced plasticity. To investigate the involvement of TRPA1 further, we used a genetic mouse model. MEP recordings from TRPA1 knockout (KO) mice showed a substantial reduction in the amplitude of MEPs evoked by a single ultrasound stimulus, indicating that TRPA1 serves as a unique sensor of LILFU. Furthermore, following iTBUS, TRPA1 KO mice exhibited a considerable decrease in LTP-like plasticity compared to littermate controls. The LTD-like plasticity induced by cTBUS initially showed a substantial increase during the first 25 min in TRPA1 KO mice. However, it eventually reached normal levels after 30 min, similar to the littermate controls (Fig. 2C). These observations highlight the crucial role of TRPA1 in mediating both LTP and LTD-like plasticity induced by TBUS.

Previous studies have indicated that astrocytic TRPA1 channels, which mediate calcium influxes, along with calcium-activated bestrophin-1 (Best1) channels, play a role in the release of signaling molecules like glutamate and d-serine, resulting in NMDAR-dependent synaptic plasticity (*40*–*42*). To explore the link between Best1 action and TBUS-induced plasticity, we examined the MEP response in M1 of both Best1 KO and littermate mice. The response profiles to iTBUS and cTBUS in Best1 littermate were consistent with the observed patterns of LTP- and LTD-like plasticity observed in WT mice; however, the after-effects induced by iTBUS and cTBUS were impaired entirely in the Best1 KO mice (Fig. 2C).

Collectively, these results support the notion that TRPA1 and Best1 are involved in TBUS-induced plasticity in M1, suggesting the essential role of TRPA1 and Best1 for NMDAR-dependent plasticity-like changes of MEP amplitudes and sensors for low-frequency ultrasound. Note that these results emphasize the nonthermal, mechanical bio-effects of iTBUS and cTBUS, which align with the findings presented in fig. S1D, illustrating that iTBUS and cTBUS stimulation did not induce any notable temperature changes.

### Role of calcium transients and NMDA receptors in ultrasound-induced plasticity

**Fig. 3.**
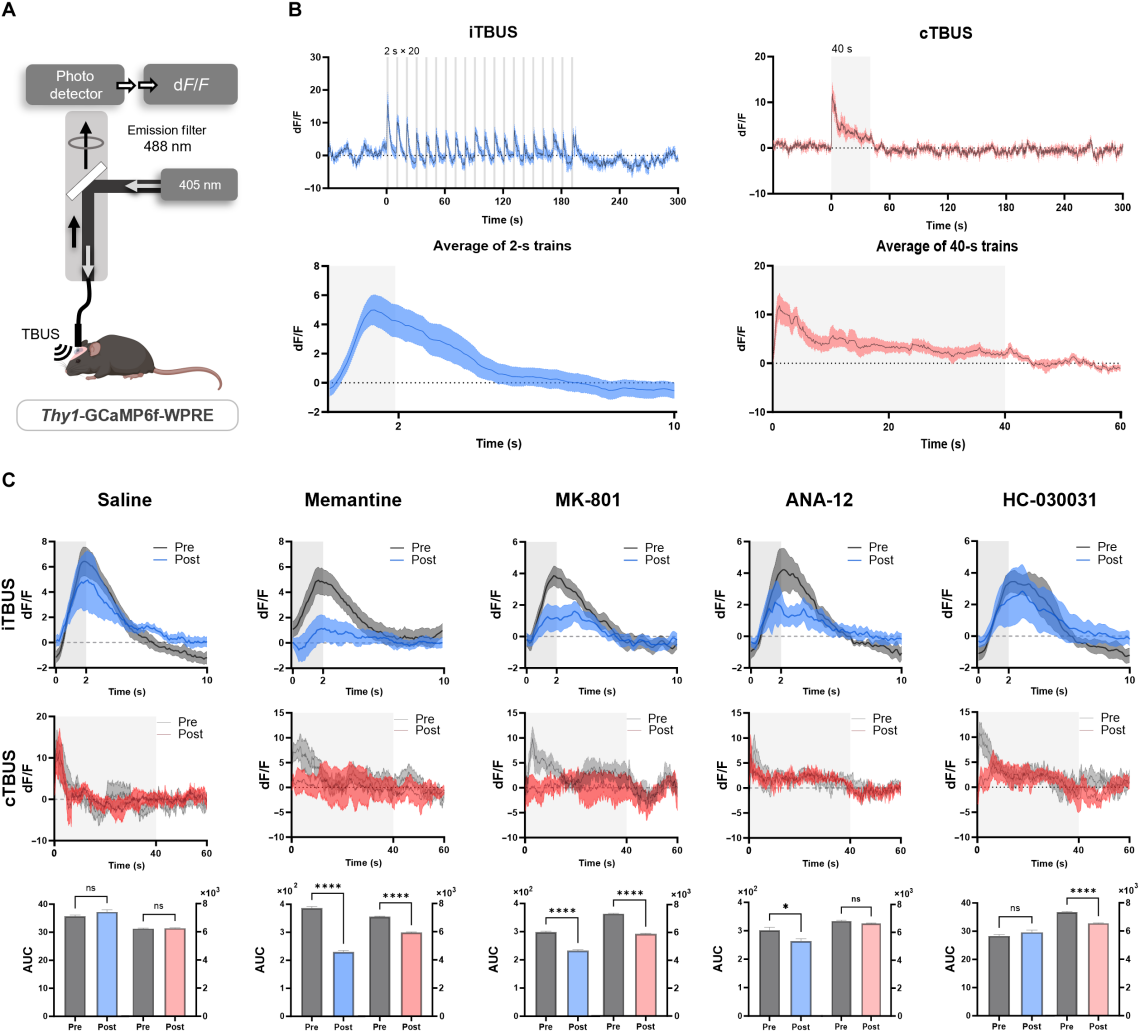
Calcium transients in M1 during iTBUS and cTBUS. (**A**) Experimental setup: GCaMP6f transgenic mice were used for neuronal calcium imaging during transcranial TBUS treatment in M1. (**B**) d*F*/*F* signal of M1 in GCaMP6f transgenic mice with iTBUS and cTBUS treatment. For iTBUS, the graph represents the averaged response of 20 oscillatory calcium transients elicited by a single train of pulses lasting for 2 s. (**C**) Group data of d*F*/*F* signal change by iTBUS and cTBUS with drug treatment. The AUC graph represents the converted data of the d*F*/*F* signal graph. The data were presented as means ± SEM. *P* values were determined by unpaired two-tailed *t* test. In the saline group, iTBUS *n* = 10, cTBUS *n* = 9; in the memantine group, iTBUS *n* = 7, cTBUS *n* = 8; in the MK-801 group, iTBUS and cTBUS *n* = 8; in the ANA-12 group, both iTBUS pre *n* = 5, post *n* = 3; and in the HC-030031 group, both iTBUS and cTBUS *n* = 7. The baseline of iTBUS AUC = −2 and the baseline of cTBUS = −10. Every *P* value is *P < 0.05 and ****P < 0.0001; ns, not significant.

Activity-dependent plasticity induction is controlled by calcium influx and calcium-triggered signaling pathways. Several studies have demonstrated a direct relationship between in vivo spiking activities and somatic calcium transients (*43*, *44*). To investigate whether the role of calcium in plasticity extends to TBUS-induced plasticity, we conducted experiments using GCaMP6f transgenic mice to visualize the dynamics of [Ca^2+^] in excitatory neurons in layer 2/3 of M1 during TBUS application (Fig. 3A).

We observed that iTBUS elicited 20 oscillatory calcium transients in response to each train of theta burst under all stimulus conditions (Fig. 3B). The calcium transients reliably occurred during the external short burst trains. At the same time, the fluorescence remained unchanged during the interstimulus intervals. On the other hand, in the case of cTBUS, an acute calcium transient with prolonged decay was observed during the application of prolonged burst train stimulation, suggesting that calcium transients and fluctuations occurred only in response to each ultrasound burst train, regardless of the ultrasound burst train length (Fig. 3B). The differences in calcium transient patterns between iTBUS and cTBUS do not depend on duration of train. Instead, they may be influenced by the properties of receptors and channels mediating calcium influx and various pumping and buffering mechanisms (*45*).

Similar to the delayed onset of MEPs in response to a single ultrasound pulse, the calcium transient induced by each theta burst ultrasound trial exhibited a delayed instantaneous rise followed by a relatively slow decay. The decay time course, approximated by a single-exponential function with a decay time constant of 1.55 to 2 s, varied in duration compared to the 2-s theta-gamma burst train.

The spatial-temporal dynamics of TBUS-evoked calcium transients are complex. Although our qualitative assessment using fiber photometry detected fluorescence changes in each burst train, we were unable to visualize specific neuronal compartments such as soma, axons, and dendrites. Whether the putative receptors and voltage-dependent channels, including L-type and N-, P/Q-type calcium channels, are required for this TBUS-evoked calcium transients remains to be determined for the cell type of interest. Nevertheless, our findings strongly indicate an essential association between ultrasound-induced plasticity and intracellular calcium transients and their oscillatory activity.

Furthermore, we compared the calcium transient between pre- and posttreatment of ion channel blockers during TBUS application. At both iTBUS and cTBUS, the inhibition of NMDARs using memantine and MK-801 resulted in a reduction of calcium transients, suggesting that the activation of NMDARs and synchronized Ca^2+^ oscillation frequency play a role in the neuroplasticity induced by both intermittent and continuous ultrasound stimulation patterns (Fig. 3C). ANA-12, a TrkB receptor antagonist, reduced calcium transient only in the iTBUS-treated group, while HC-030031, a selective TRPA1 inhibitor, showed its effect only in the cTBUS-treated group (Fig. 3C). These findings suggest the selective roles of TrkB and TRPA1 for perceiving each patterned ultrasound stimulation.

### Role of TBUS in enhancing neuronal activity and AMPA receptor plasticity in the motor cortex

**Fig. 4.**
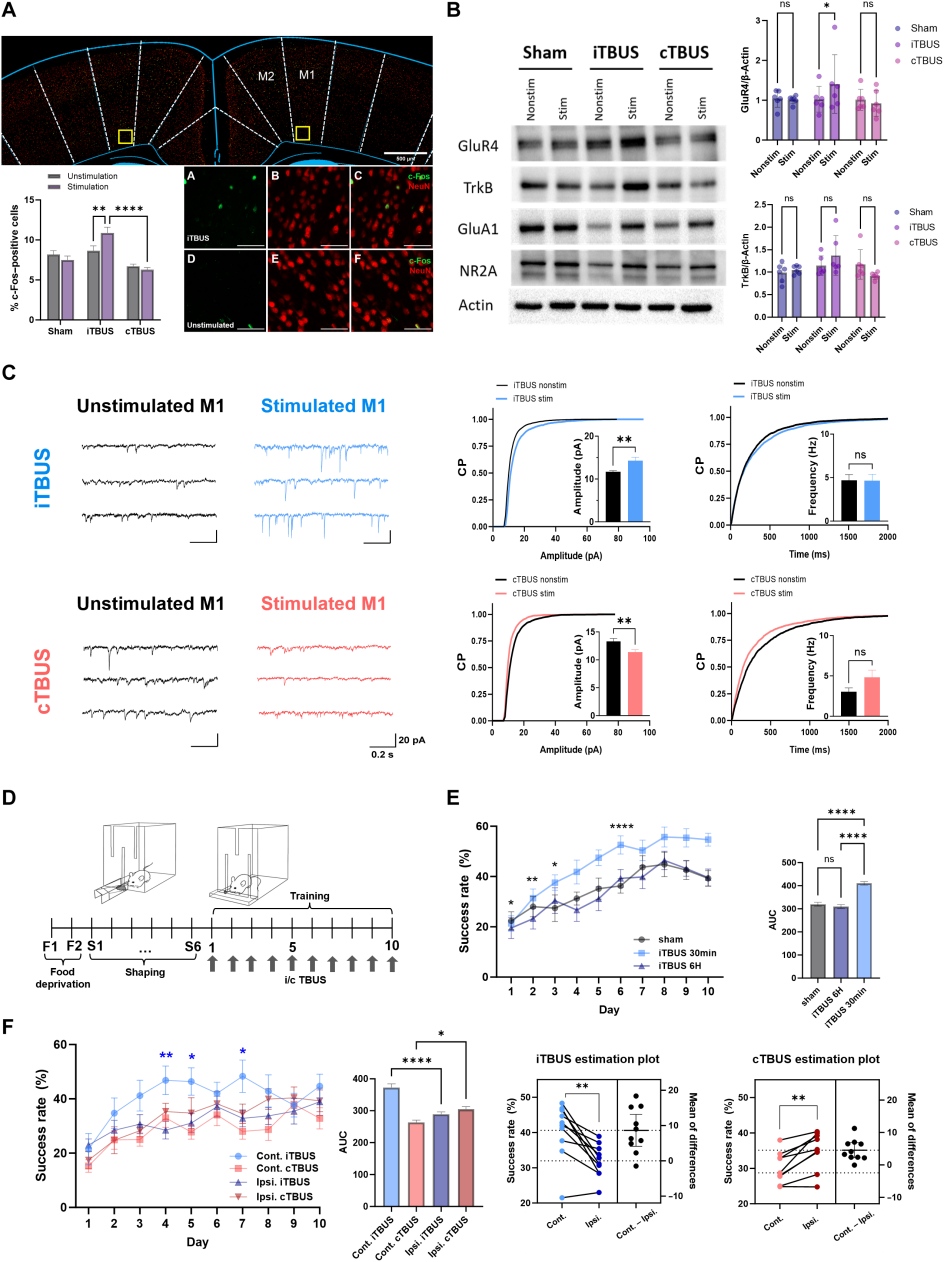
TBUS effects on neuronal activity, synaptic plasticity, and skill learning in mice. (**A** and **B**) Representative c-Fos immunofluorescence images in M1, with data presented as means ± SD (*n* = 7 for each group). Western blots data in the left M1 (*n* = 6 for each group). Two-way ANOVA and Šidák’s multiple comparison tests were used. The *P* values are indicated as *P = 0.03, **P = 0.0038, and ****P < 0.0001. (**C**) Representative whole-cell patch-clamp recording traces and cumulative probability distribution of spontaneous excitatory postsynaptic currents. An unpaired two-tailed *t* test was used for analysis. (**D**) Experimental setup for the single-pellet reaching task (SPRT). (**E**) Success rates over 10 days are based on the timing of stimulation. Comparison between iTBUS applied 30 min before training and iTBUS applied 6 hours before training. Unpaired *t* test with the calculation of AUC and one-way ANOVA with Tukey’s test were used for analysis. (**F**) Success rates of the SPRT (*n* = 9 for each group). An unpaired two-tailed *t* test and one-way ANOVA with Tukey’s test were used for analysis. The *P* values are indicated as *P < 0.05, **P < 0.01, and ****P < 0.0001, or ns, not significant, in the estimation plot.

The expression of immediate early gene c-Fos plays a crucial role as a molecular marker for specific subsets of neurons undergoing plastic changes during long-term memory formation. In our study, we aimed to investigate the effect of TBUS on neuronal activity in M1 and its effect on neuronal excitability (*46*). Following iTBUS, we observed that c-Fos expression was highly increased in the stimulated M1 than in the nonstimulated M1 (Fig. 4A). However, no difference in c-Fos expression was observed between the hemispheres that received cTBUS and the sham group (Fig. 4A). These findings are consistent with previous reports highlighting the involvement of c-Fos in LTP (*47*, *48*).

To further assess the effect of TBUS on plasticity, we examined the expression of α-amino-3-hydroxy-5-methyl-4-isoxazolepropionic acid receptor (AMPAR) subunits as markers of plasticity (*46*). Western blot analysis revealed that the glutamate receptor 4 (GluR4) AMPAR subunit was highly expressed following iTBUS (1.026 ± 0.29 in nonstimulated M1 and 1.408 ± 0.67 in stimulated M1, *P* = 0.03) (Fig. 4B). This finding aligns with previous evidence suggesting the association of GluR4 with LTP (*49*).

Collectively, these results demonstrate that iTBUS can effectively enhance c-Fos expression and induce the up-regulation of the AMPAR subunit GluR4, suggesting a potential role of TBUS in modulating neuronal activity and synaptic plasticity in M1.

We conducted electrophysiological recordings to further investigate the effects of iTBUS and cTBUS on synaptic AMPARs. Whole-cell patch-clamp recordings of pyramidal neurons in M1 revealed that the average amplitude of spontaneous excitatory postsynaptic currents (sEPSCs) was highly increased following iTBUS application compared to nonstimulated M1 (stimulated: 14.29 ± 3.50 pA; *n* = 20 cells; nonstimulated: 11.70 ± 1.01 pA; *n* = 19 cells; *P* < 0.0001) (Fig. 4C). This suggests an enhancement of synaptic AMPAR-mediated neurotransmission in response to iTBUS. Conversely, cTBUS application resulted in a decrease in sEPSC amplitude (stimulated: 11.39 ± 1.52 pA; *n* = 12 cells; nonstimulated: 13.33 ± 1.68 pA; *n* = 12 cells; *P* < 0.001) (Fig. 4C). In addition, cTBUS application led to an increase in the frequency of sEPSCs (stimulated: 4.83 ± 3.03 Hz; *n* = 12 cells; nonstimulated: 3.03 ± 1.67 Hz; *n* = 12 cells; *P* < 0.05) (Fig. 4C). These findings indicate a reduction in synaptic AMPAR-mediated neurotransmission following cTBUS. Overall, the cumulative histograms of sEPSCs demonstrate that TBUS produces selective alterations in synaptic AMPARs, indicating that theta-gamma patterned ultrasound stimulation can induce synaptic plasticity. An intriguing observation is that patterned TBUS stimulation did not strongly affect the neuronal excitability in the M1 motor cortex (fig. S10). In conclusion, under the experimental conditions that we used, it can be said that theta-gamma patterned ultrasound stimulation influences synaptic plasticity.

### Modulation of behavioral learning by TBUS in the motor cortex: Role of iTBUS

LTP- and LTD-like plasticity has been observed in M1 while learning a new motor skill (*50*). For the translational use of TBUS, it is critical to investigate whether TBUS could modulate or interact with behavioral learning. To assess the contribution of TBUS, we conducted experiments using a single-pellet reaching task (SPRT) in healthy WT mice. The SPRT involved presenting a food pellet behind a transparent wall, requiring the mice to reach for the food pellet with one of their forelimbs through a narrow slot (*51*). On each day of the training session, we applied either 800 pulses of iTBUS or cTBUS over either M1 contralateral and ipsilateral to the performing forelimb in a 2 × 2 factorial design 30 min before training (Fig. 4D). iTBUS and cTBUS over the ipsilateral M1 to the performing forelimb served as active control conditions for possible nonspecific effects, such as arousal and auditory startle response (*24*, *25*).

LTP-like MEP changes induced by iTBUS 800 in M1 lasted for nearly 1 hour (Fig. 2A). We investigated the effects of iTBUS administered 30 min and 6 hours before SPRT training. iTBUS delivered to the contralateral M1 6 hours before had no critical impact on the SPRT learning curve, while iTBUS administered 30 min prior shifted the curve to the left, indicating enhanced incremental trial and error learning in SPRT (Fig. 4E).

In the contralateral stimulation conditions, the success rate in acquiring the motor skill was higher for iTBUS compared to cTBUS (40.60 ± 2.14% versus 28.75 ± 1.96%, *P* = 0.0016), while no notable difference was observed between groups in the ipsilateral conditions (Fig. 4F). Specifically, iTBUS over the contralateral M1 led to increased skilled reaching performance compared to iTBUS applied to the ipsilateral M1 (40.60 ± 1.96% versus 31.97 ± 2.39%, *P* = 0.008), demonstrating that iTBUS specifically facilitated the acquisition of the new motor skill (Fig. 4F).

The averaged learning curve comprised an early acquisition phase with progressive success rate improvement and a later consolidation phase after the success rate reached the plateau level. In this study, the learning curve analysis showed that the iTBUS-treated group exhibited a left-shifted and increased success rate over time compared to the active control and cTBUS group (Fig. 4F). These findings differ from a previous study showing that the range of synaptic modification is not affected by motor skill learning, and synaptic changes reach the plateaus within the range of synaptic modification (*50*). These results highlight the importance of timing for “priming” stimulation in facilitating motor skill learning (Fig. 4E), consistent with studies using TMS and transcranial electrical stimulation (TES) for M1 priming stimulation to improve subsequent motor skill acquisition (*52*). To rule out the possibility that the reduced success rate in the contralateral cTBUS group was due to reduced motor activity, similar to the case of motor cortex injury (*53*), we examined the number of reaching attempts per minute. We found that the cTBUS group made more attempts in the contralateral conditions than the iTBUS group (fig. S8). Therefore, it is possible that the increased number of attempts was a compensatory mechanism for poor motor skills following cTBUS.

To ensure the safety of iTBUS and cTBUS, we conducted hematoxylin and eosin staining to detect any possible hemorrhaging or tissue damage after 10 days of TBUS administration. In addition, we administered Evans blue (EB) dye to assess the potential disruption of the blood-brain barrier following the acute administration of 800 TBUS pulses. The results showed that TBUS did not induce any tissue damage or alterations in EB distribution, indicating the absence of gross tissue changes when TBUS was applied using an ultrasound intensity relative to the motor threshold (fig. S9). Overall, these findings suggest that TBUS has the potential to modulate behavioral learning, as iTBUS applied to the contralateral M1 specifically enhanced the acquisition of a new motor skill.

## DISCUSSION

To date, investigation into the effects of noninvasive techniques such as TMS or TES on humans and animals has primarily relied on measuring changes in motor output strength, as reflected in MEP amplitude (*13*, *14*). In this study, we used the same methodology to assess the robustness and longevity of our patterned TBUS coupled with gamma rhythms, which offers advantages in terms of high spatial resolution and targeted depth compared to other noninvasive methods.

Our findings revealed that MEP amplitude can be either facilitated or suppressed depending on the pattern of TBUS delivery, specifically intermittent versus continuous stimulation. We confirmed that theta burst patterned delivery of ultrasound has longer-lasting effects compared to single-frequency stimulation at 10 Hz, and theta stimulation with the “gamma bursting” pattern is more effective than regular theta stimulation, even when the total energy delivered is identical (fig. S6). Notably, we observed that the facilitation duration of iTBUS 400 pulses can be twofold when using 800 pulses (Fig. 2A). This phenomenon is not achievable with magnetic theta burst stimulation (*12*, *28*). Hence, iTBUS may provide a unique opportunity to treat brain disorders characterized by reduced excitability and neuroplasticity, such as depression.

Our study demonstrates that TBUS-induced plasticity effects require NMDAR activation, BDNF/TrkB signaling, and de novo protein synthesis, commonly required for inducing a longer-lasting form of plasticity known as late LTP and LTD (*54*). In addition, we found that TRPA1 and Best1 play an essential role in TBUS-induced plasticity of MEP amplitude, representing a critical function of ultrasound neuromodulation for NMDAR-dependent neural plasticity. These findings highlight the involvement of TRPA1 and Best1 ion channels in TBUS-induced plasticity, distinguishing it from electrical stimulation-induced plasticity while sharing the same molecular mechanism underlying synaptic plasticity. Furthermore, we observed that iTBUS delivered over the contralateral M1 facilitated the acquisition of a new motor skill related to reaching and grasping. In contrast, the same effect was not observed with ipsilateral delivery as active controls (Fig. 4F). This hemispheric specificity aligns with evidence indicating that skilled reaching training strengthens layer 2/3 horizontal synaptic connections in M1 corresponding to the trained forelimb but not in M1 corresponding to the untrained forelimb (*50*, *55*). Thus, the TBUS-induced LTP/LTD-like phenomena observed in MEPs may directly contribute to the functional interactions with training-induced LTP during the early stages of motor skill learning.

In conclusion, our study has demonstrated the effectiveness of patterned ultrasound-induced brainwave entrainment in inducing long-lasting plasticity changes in the motor cortex. With the unique ability to precisely focus ultrasound waves on specific deep brain regions, patterned transcranial ultrasound stimulation holds promise for safely producing predictable and enduring changes in brain function. Ultrasound waves primarily affect neural circuits by influencing mechanosensitive ion channels and receptors (*56*, *57*). We cannot exclude the possibility that ultrasound-evoked mechanical force activates NMDARs and mechanosensitive ion channel component 1 (Piezo1) as mechanosensitive ion channels in an agonist-independent manner (*58*–*60*). Our study provides compelling evidence supporting the effectiveness of a robust ultrasound protocol in inducing long-lasting plasticity changes in the motor cortex, laying the foundation for safe and predictable alterations in brain function. The continuous interaction of TBUS with subsequent motor skill acquisition suggests its translational potential for human applications. These ultrasound parameters can also be adapted for other brain stimulation protocols, such as evoking sensory-evoked potentials or visual-evoked potentials. Moreover, exploring PAC and cross-frequency coupling in specific brain regions and frequencies offers promise for long-lasting neuromodulatory effects in future research.

## MATERIALS AND METHODS

### Animals

C57BL/6 and Thy1-GCaMP6f strain mice were used at different ages (8 to 14 weeks) in the study. All experiments reported in this study were approved by the Institutional Animal Care and Use Committee at the Institute for Basic Science in South Korea.

### Ultrasound experimental setup and stimuli

Two essential aspects of our protocol should be noted here. After conducting pilot testing with various schemes (e.g., figs. S3, S4, and S6), we cross-coupled a slow gamma frequency of 30 Hz with a theta cycle of 5 Hz (TBUS; see also fig. S3). This maximized sonication time within the slow gamma range, aiming for an approximately 50% duty cycle at 30 Hz. The sonication duration is an essential factor in determining ultrasound-induced neural effects following the buildup to a threshold (or accumulation) hypothesis (*22*). The other important aspect of our protocol was the determination of ultrasound intensity for TBUS. We referenced ultrasound intensity to the threshold of motor response. This approach is well established in the TMS literature that the intensity for theta burst stimulation (also for other plasticity induction protocols) should be referenced to the intensity that produces a motor response (i.e., 80 to 90% of motor threshold; subthreshold stimulation). Too much or too little intensity would not produce any notable plasticity effects (*9*, *38*).

Theta burst ultrasound waveforms were generated using a pulse generator (Master 9, AMPI, Jerusalem, Israel) and two function generators (www.keysight.com/). The Master 9 generated gamma-nested theta TTL (transistor-transistor logic) pulses to function generator 1. During iTBUS, four TTL pulses were delivered at 30 Hz (33.33-ms interstimulus onset interval), and this pattern was repeated at 5 Hz (200 ms) for a duration of 2 s, with an inter-train interval of 8 s. In cTBUS, the 2 s of TBUS was delivered continuously without the inter-train interval of 8 s. Upon each TTL pulse from the Master 9 pulse generator, function generator 1 produced 17 pulses at a PRF of 1 kHz, resulting in a pulse duration of 17 ms. The output of function generator 1 served as an external trigger for function generator 2 to generate 500 cycles of sine waves per PRF (duty cycle 100%) at an acoustic frequency of 500 kHz, totaling 8500 cycles. For the 30-Hz gamma pattern using four 17-ms pulses, these four pulses (totaling 68 ms) are delivered over a period of 133.2 ms, resulting in a duty cycle of 51.1%.

The resulting sinusoidal waves were amplified using a 75-W RF power amplifier (A075, E&I, Rochester, NY, USA) and delivered to a transducer (Olympus Panametrics V301). The transducer used for tFUS stimulation in the M1 cortex had a diameter of 25 mm and operated at a center frequency of 500 kHz. It was equipped with a cone-shaped acoustic collimator filled with ultrasound gel (Pro-gel II, Dayo Medical, South Korea) to focus the ultrasound energy precisely. The transducer specifications included an ultrasound aperture outer diameter (OD) of 25 mm, an ultrasound fundamental frequency (f0) of 0.5 MHz, and a −6-dB bandwidth ranging from 350 to 640 kHz. The focal depth of the transducer was adjustable within the range of 31.75 to 41.91 mm (V301-SU, Olympus Scientific Solutions Americas Inc., USA). The collimator length was adjusted and measured using a needle hydrophone system to ensure that the ultrasound stimulation was delivered effectively within a specific range from the collimator outlet (Precision Acoustics, UK). The collimator, 3D-printed to match the transducer’s focal length and the animal model’s dimensions, featured a circular outlet with an area of 4.91 mm^2^ (radius: 1.25 mm). This adjustment was made to limit the ultrasound stimulation to a precise depth, allowing for the stimulation of the shaved scalp (~0.5 mm), skull bone (~0.5 mm), and cortex (~1 mm) from the collimator outlet or within a similar range. It ensured that the ultrasound stimulation was effectively delivered within a 2-mm range from the end of the ultrasound collimator (see fig. S1). For MEP measurement, we stimulated the left motor cortex with the same U.S. parameters (center frequency = 500 kHz, PRF = 1 kHz with 17 cycles, and duty cycle = 100%) to produce reliable limb movements. Two stable baseline MEP blocks (10 min each) were conducted just before TBUS, followed by 6 or 10 post-TBUS blocks lasting 50 min. During each 5-min block, MEPs were obtained by delivering 25 ultrasound stimuli with jittered intervals of approximately 10 to 15 s.

Ultrasound intensity was measured at the end of the acoustic collimator using a needle hydrophone system (Precision Acoustics, UK). For the 17-ms ultrasound stimulus for eliciting MEPs, the spatial-peak pulse-average intensity (*I*_sppa_) was 2.787/cm^2^, using an equation of *A*2/2ρ*c*, where *A* is the peak rarefactional pressure (0.299 MPa), ρ is the density of the medium (1028 kg/m^3^), and *c* is the speed of sound in the medium (1515 m/s), as calculated in previous studies (*40*). The mechanical index (MI), representing the ultrasound beam’s bio-effect (MI = *A*/√*f*), was 0.422. The same stimulus was used in TBUS, but its intensity was reduced to 80%. The spatial-peak temporal-average intensity (ISPTA) for iTBUS was 54.4 mW/cm^2^, and ISPTA for cTBUS was 272 mW/cm^2^. They are well below the U.S. Food and Drug Administration clinical ultrasound imaging thresholds (MI = 1.9 and ISPTA = 720 mW/cm^2^). Note that the root mean square value of pressure was used in all the above calculations.

### Ultrasound beam profile and temperature measurement

The acoustic beam profile of the transducer (V301-SU, Olympus Scientific Solutions Americas Inc., USA) was measured in a water tank using a needle hydrophone with a 2-mm diameter (NH2000, Precision Acoustics Ltd., UK). The radio frequency (RF) amplifier used for this purpose was a 50-W RF power amplifier (350 L, E&I Rochester, NY, USA). A needle hydrophone was connected to a motorized XYZ stage, and the transducer, integrated with a collimator, was fixed in the transducer holder. To accurately measure the ultrasound beam profile delivered into the mouse brain, axial and radial acoustic beam profiles were obtained with a step size of 100 μm in both free-field (water) and with a dissected skull. Because of the potential risk of damaging the surface of the hydrophone during scanning, the nearest distance at which we could measure the beam profile was 1 mm in both situations. The input voltage, acoustic frequency, and number of cycles were 200 mVpp, 500 kHz, and 3 cycles, respectively. To measure the temperature increase in both iTBUS and cTBUS stimulation conditions, a needle thermometer (K-type, Misumi) was combined with an ex vivo mouse brain. The needle thermometer was inserted vertically, with its tip in the mouse brain cortex. To simulate an in vivo setting during stimulation, the mouse skull was positioned at the surface of the mouse brain, and the transducer, integrated with a collimator, was placed vertically above the surface of the mouse skull. All of these measurement configurations were conducted in a water tank filled with distilled water, and the water temperature was maintained using a hot plate throughout the experiment. A temperature controller (VX4, Hanyoung Nux) was used to record the temperature readings from the thermometer.

### MEP experiments and data analyses

The animal was anesthetized in an induction chamber using 2% isoflurane (Piramal Critical Care, Bethlehem, PA, USA) delivered with oxygen at a flow rate of 1.5 liters/min using an isoflurane anesthesia system (Harvard Apparatus, Holliston, MA). The eyes were covered with ophthalmic ointment to keep them moist. The hair on the dorsal surface of the head, where the ultrasound transducer would be placed, was trimmed using clippers, and depilatory cream was then applied (Nair, Church & Dwight, Frenchs Forest, Australia). Subsequently, the animal was immobilized using a stereotactic instrument with the head fixed using ear bars, while the forelimbs were suspended. At the start of the experiment, an isoflurane level of 0.25% delivered via a nose cone served as the anchor point to achieve an individually tailored light anesthetic state. This adequacy of anesthesia was confirmed by observing pinch responses. Once the appropriate anesthetic level was determined, the same isoflurane level was maintained throughout the experiment.

The elicited limb movements were recorded using fine wire electrodes (A.M. Systems, catalog no. 790900, Sequim, WA) and PowerLab 26 T (AD Instruments). Two pairs of fine wire electrodes were inserted approximately 3 to 5 mm apart along the right triceps brachii to record the bioelectric potential difference across the muscle tissue. A common ground electrode (Grass Technologies, F-E2) was inserted subcutaneously into the back. Electromyography signals were sampled at 2 kHz. To elicit MEPs from the triceps brachii of the forelimb through 0.5-MHz ultrasound stimulation of the M1 cortex, a stimulation duration ranging from a minimum of 3 to 20 ms is required. In this study, the 17-ms duration was chosen as it reliably induced MEPs. Raw MEPs were subjected to band-pass filtering within the 10- to 500-Hz range, followed by rectification and smoothing with a zero-phase lag second-order Butterworth filter with a cutoff frequency of 20 Hz. The peak amplitude of the resulting linear envelope represents motor cortex excitability. A 50-ms window (100 samples) just before the onset of the ultrasound stimulus served as a baseline. We used the integrated profile for MEP onset times because the widely used 3σ threshold approach does not accurately capture MEP onset times when the signal-to-noise ratio is low (contaminated with cardiac signals). Conceptually, the IP method detects the starting point of sharp slope increase using the cumulated area of rectified signal over time (see our comparisons for different methods in fig. S5). Throughout the experiments, we recorded five MEPs per minute, acknowledging the inherent variations within MEPs during offline analysis. Data were processed and presented by aggregating the results in 5-min intervals using grand averages for measuring MEP amplitude and onset latency. Subsequently, a comprehensive comparative analysis was carried out on the basis of these measurements.

### MATLAB toolbox for estimating PAC

We used EEGlab and PAC code by https://doi.org/10.5281/zenodo.10462520 in MATLAB. This code modifies the open-Ephy data to the PAC graph.

###  In vivo fiber photometry

Thy1_GCaMP6f mice were fixed using stereotaxic (using multi-function adaptors #68091, RWD) and anesthetized with 2% isoflurane for laminectomy. After the scalp incision and clearance of surrounding tissues with a cotton swab, only the skull remains. Using a bent insulin syringe needle, bent at a 90° angle, the 2-mm square windows with lambda as one vertex on the left and right parietal bones are made. For stability, the mouse is placed on a 38°C heat pad with a soft cushion. For ultrasound stimulation, the ultrasound transducer is positioned over the M1 cortex. An optical cannula is placed over the ultrasound transducer stimulation site for recording. Anesthesia level is reduced from 2 to 0.5% during recording. Calcium responses are observed for every iTBUS and cTBUS train. Light-emitting diodes at 488 nm (GCaMP6f stimulation wavelength) and 405 nm (control for artifactual fluorescence) (Doric) were coupled to a 400-μm 0.4 NA 0.22 optical fiber (ZF1.25, Doric). The same optical fiber collected the emitted light passed through a green fluorescent protein filter and focused onto a fluorescence detector (fluorescence mini cube, Doric), where the two output signals were separated on the basis of modulation frequency. Samples were collected at a frequency of 330 Hz. The recording consists of a baseline period of 60 s before stimulation, followed by ultrasound stimulation (iTBUS: 200 s; cTBUS: 40 s) and a post-stimulation period (iTBUS: 120 s; cTBUS: 260 s) for a total duration of 300 s. For drug administration, with the head immobilized, the right hind limb is lifted to perform an intraperitoneal (ip) injection, followed by a waiting period of 15 to 30 min.

Data were extracted and analyzed using custom-written scripts. To normalize the data, the control channel was fitted to and then subtracted from the raw trace, resulting in the calculation of Δ*F*/*F*.

### Pharmacological experiments

To investigate whether TBUS-induced plasticity effects were NMDAR-dependent, the animals were randomly assigned to three experimental groups (six animals in each group) for iTBUS and other three groups for cTBUS to receive either an acute injection of memantine (Tocris, Bristol, UK) at the dose of 5 mg/kg (6.25 ml/kg, ip), DCS (Sigma-Aldrich, St. Louis) at a dose of 3 mg/kg (10 ml/kg, ip), or saline (0.9% NaCl, ip). As MK-801 has a higher affinity for NMDARs than memantine, we used MK-801 (Tocris, Bristol, UK) at a dose of 0.1 mg/kg to further test the NMDAR dependency of TBUS-induced plasticity effects. To examine whether TBUS induces de novo protein synthesis, the animals were intraperitoneally injected with CXM (Sigma-Aldrich, St. Louis) in a 30 mg/kg (10 ml/kg, ip) dose or saline. To examine whether BDNF signaling produces TBUS-induced after-effects, we administered ANA-12 (0.5 mg/kg) (dissolved in 1% DMSO in saline, 10 ml/kg, ip) or 1% DMSO in saline. To examine whether the TrpA1 ion channel is involved in producing TBUS-induced after-effects, we administered HC-030031 (100 mg/kg) dissolved in 5% DMSO and 10% Tween 80 in saline [or HC-030031 (20 mg/kg) dissolved in 1% DMSO and 10% Tween 80 in saline].

### Single-pellet reaching task

We used the SPRT, as mice are not naturally adept at this task, to test our hypothesis that iTBUS facilitates, and cTBUS impedes, the learning of this new motor skill. We used an experimental chamber as previously described (*51*). The acrylic chamber was 20 cm tall, 15 cm deep, and 8.5 cm wide, with a vertical slit (0.5 cm wide) downward and two vertical slits (two 0.5-cm-wide splits with 2.5-cm distance) upward on the front. The chamber was used for both shaping (single slit side facing downward with a pellet tray in the front) and training (two slit sides facing downward with a pellet platform with two indentations for pellets corresponding to the two slits).

From 2 days before the start of shaping, food was restricted to 10% of the body weight per day until the end of the experiment. During shaping, animals were habituated to the chamber. Pellets (20 mg of grain pellet, product # F0163, Bio-Serv, NJ) were placed on the tray in front of the center slit, allowing the animal to retrieve pellets with both forelimbs. Shaping was finished when the animal made 20 reaching attempts within 20 min. Forelimb dominance was determined by the use of one forelimb in more than 70% of the attempts. Animals not reaching the criteria within 8 days of shaping were excluded. After shaping, animals underwent a single pellet-reaching training session for 10 consecutive days. A training session finished after 30 reaches or 20 min of training, whichever was achieved first. We applied either 800 pulses of iTBUS or cTBUS over the contralateral or ipsilateral motor cortex to the performing forelimb before the training session each day. In a 2 × 2 factorial between-subjects design (TBUS type × hemisphere), nine animals were included in each group. Animals were allocated to one of four experimental groups after stratifying for a number of days required to finish shaping and forelimb dominance (fast learner: 1 to 2 days; intermediate learner: 3 to 5 days; slow learner: 6 to 7 days; see table S1). Each session was video-recorded to refer to the online scoring.

Reaching behavior was scored as a success (if the pellet was grasped, retrieved, and then placed into the mouth using the dominant forelimb), a fail (if the pellet was reached but not grasped, or if the pellet was grasped but then dropped, with the dominant forelimb), or a miss (an unsuccessful attempt to reach the pellet with the dominant forelimb). Misses were categorized as fails.

### Immunohistochemistry

We applied 800 pulses of iTBUS, cTBUS, or sham stimulation over the left M1. Following 30 min after TBUS, they were deeply anesthetized using ketamine and xylazine injection (0.13 g/kg, ip). The animals were then transcardially perfused with phosphate-buffered saline (PBS; pH 7.4), followed by 4% paraformaldehyde in PBS. After this, the brains were extracted, postfixed in 4% paraformaldehyde at 4°C overnight, and immersed in a solution of 30% sucrose in PBS at 4°C until they sank. Brains were sectioned in the coronal plane at 20 μm around the motor cortex (Leica VT 1200s, Germany). The slices were immune-labeled at 4°C overnight using antibodies against c-Fos (1:3000; sysy226003, Synaptic Systems, Goettingen, Germany) and NeuN (1:500; Millipore, catalog no. MAB377). After this, the slices were washed with 1× PBST (0.3% Triton X-100 in 1× PBS) and incubated at room temperature for 2 hours in secondary antibody [1:800; Goat Anti-Rabbit IgG (H&L), Alexa Fluor 568, Abcam, ab175471 and 1:800; CyTM5-conjugated AffiniPure Goat Anti-Mouse IgG (H&L), Jackson ImmunoResearch, 115-175-146]. The slices were then washed with 1× PBST and mounted on slide glass. Two-channel confocal images were acquired using a confocal microscope (an inverted research microscope Nikon Eclipse Ti-E with the Perfect Focus System, Nikon Instruments Inc., Tokyo, Japan). The resulting images were processed and analyzed using ImageJ (https://imagej.nih.gov/ij/).

### Electrophysiology: Slice preparation and sEPSC recordings

Twenty minutes after the application of TBUS 800 pulses, the mice were anesthetized with isoflurane. Coronal M1 slices (280 μm) were dissected and placed immediately into the cold (2.5°C) modified cerebrospinal fluid (CSF) containing (in millimolar) 205 sucrose, 2.5 KCl, 7 MgCl_2_, 0.5 CaCl_2_, 1.25 NaH_2_PO_4_, 3 pyruvate, 1.3 ascorbic acid, 25 sodium bicarbonate, and 7 glucose, equilibrated with 95% O_2_ and 5% CO_2_ (pH 7.4, 305 ± 5 mmol/kg). After cutting, the slices were incubated for 15 min at 32°C and then for up to 3 hours at 28°C in artificial CSF.

Whole-cell patch-clamp recordings from slices were carried out at 25° to 28°C. Pyramidal neurons in layers II/III of M1 were visually identified using Dodt Gradient Contrast. The recording chamber was continuously perfused with artificial CSF containing (in millimolar): 124 NaCl, 2.5 KCl, 1.3 MgCl_2_, 2.5 CaCl_2_, 1 NaH_2_PO_4_, 26.2 NaHCO_3_, and 10 glucose, equilibrated with 95% O_2_ and 5% CO_2_ (pH 7.4, 305 ± 5 mmol/kg). The pipette solution contained 127.5 mM cesium methanesulfonate, 7.5 mM CsCl, 10 mM Hepes, 2.5 mM MgCl_2_, 4 mM Na_2_ATP, 0.4 mM Na_3_GTP, 10 mM sodium phosphocreatine, and 0.6 mM EGTA (pH 7.25). Patch pipettes were pulled from borosilicate glass (4 to 7 megohms) using a horizontal puller (Sutter Instruments, Novato, CA). Signals were recorded with the MultiClamp 700B (Molecular Devices, Union City, CA) amplifier and sampled at 10 kHz. To detect sufficient events (200 events per neuron), recordings were performed in gap-free mode (sweeps of 30 s without any gap). Data were acquired 3 min after achieving whole-cell configuration. Series resistances (*R*_s_) of recordings ranged between 10 and 15 megohms. Cells were excluded from the analysis if the *R*_s_ changed by more than 15%. sEPSCs were analyzed by Mini Analysis Software (Synaptosoft, NJ). All group data are shown as means ± SEM. Statistical comparisons were performed by the independent *t* test. All drugs were purchased from Sigma-Aldrich.

### Extracellular electrophysiology

Electrophysiological recordings were made with Neuropixels probes (Phase 3B1) (*61*) in the motor cortex (M1) of head-fixed and anesthetized mice, while ultrasound stimulation was given. Recordings were made from the left hemisphere. A small craniotomy (1-mm diameter) was made over the recording sites (A/P 0.3 mm, M/L 1.25 mm) 30 min before the recording session. The probe was attached to a four-axes micromanipulator (Luigs & Neumann) and lowered slowly (2 to 8 μm/s) to a maximum of 1 mm from the dura with a 45° tilt. After reaching the desired depth, the probe was allowed to settle for 10 min before the commencement of recording. The collimator was positioned 100 μm above the craniotomy, perpendicular to the cortex surface. The space between the collimator and the brain was filled with PBS, and the external reference for the Neuropixels probe was immersed within the PBS. Recordings were made with the open-source software SpikeGLX (http://billkarsh.github.io/SpikeGLX/). Recording depth was inferred from manipulator readings. The data were automatically spike sorted with KiloSort2 (*62*) (https://github.com/cortex-lab/Kilosort/) and then manually curated with the phy GUI (https://github.com/kwikteam/phy/). We used potentially suitable units for analysis: amplitude > 100 μV, firing rate > 0.2 Hz, SNR > 2.5 throughout recording sessions. We simply divided recorded single units into two clusters by trough-to-peak duration: broad-spiking (trough-to-peak latency > 400 μs) and narrow-spiking (trough-to-peak latency < 400 μs) neurons.

### Western blot analysis

Brain tissues were extracted from the left motor cortex 1 hour after iTBUS 800, cTBUS 800, or sham stimulation. They were homogenized in a homogenization buffer containing 1× PBS, 0.303 M sucrose, and a 25× protease inhibitor cocktail (cOmplete, ref 11873580001). Homogenates were centrifuged at 1920*g* at 4°C for 10 min. The supernatants were centrifuged at 20,766*g* at 4°C for 20 min. The pellets were lysed with solubilization buffer (1× PBS with 10% TriX-100, 0.5 M EDTA, 0.5 M EGTA, 10% SDS buffer with 25× protease inhibitor cocktail) and rotated at 4°C for 2 hours to ensure complete lysing. Protein concentration was determined by BCA assay (Pierce BCA Protein Assay Kit, ref. 23228). Equal amounts (10 to 25 μg) of protein and 5× SDS sample buffer were added to the sample lysates and boiled at 95°C for 10 min. SDS–polyacrylamide gel electrophoresis was used with 5% stacking gels and 7 to 9% resolving gel, and then transferred to a polyvinylidene fluoride membrane. The membrane was blocked in a 1% solution of bovine serum albumin containing 10% sodium azide at room temperature for 1 hour and subsequently incubated with primary antibody (GluR4, 1:1,000, Thermo Fisher Scientific, catalog no. PA5-24217; GluA1, 1:1000, Abcam, catalog no. ab76321; NR2A, 1:1000, Abcam, catalog no. ab133265; TrkB, 1:1000, Abcam, catalog no. ab18987; CREB, 1:1000, Cell Signaling Technology, catalog no. 9197s) overnight at 4°C in blocking solution. The blots were washed with PBS-T (4 × 15 min) and then incubated with anti-rabbit horseradish peroxidase (HRP)–conjugated secondary antibody (1:10,000; Cell Signaling Technology, catalog no. 8457 s) for 1 hour at room temperature. After washing with PBST, the blots were developed using a western ECL substrate (SuperSignal West Atto Ultimate Sensitivity Substrate), and images were taken with sequential exposure times (ChemiDoc, Bio-Rad). The blots were stripped with stripping buffer containing 0.5 M (pH 6.7) tris-HCl and 20% SDS at 55°C for 35 min, followed by washing with distilled water for 2 hours. In a blocking solution, the blots were subsequently incubated with rabbit anti–beta-actin (1:10,000; Cell Signaling Technology, catalog no. 8457s). Goat anti-rabbit HRP-conjugated secondary antibodies were applied and again exposed to ECL substrate.
