# Supplementary material for: Long-lasting forms of plasticity through patterned ultrasound-induced brainwave entrainment
Source: Sci Adv. 2024 Feb 23;10(8):eadk3198. doi: 10.1126/sciadv.adk3198 (PMC10889366; doi:10.1126/sciadv.adk3198)
Supplement: Supplementary file 1 — Figs. S1 to S11 Table S1 Legends for movies S1 to S3 [file sciadv.adk3198_sm.pdf]

Science Advances
AAAS

Supplementary Materials for

Long-lasting forms of plasticity through patterned ultrasound-induced brainwave entrainment

Ho-Jeong Kim *et al.*

Corresponding author: Joo Min Park, joominp@ibs.re.kr

Sci. Adv. **10**, eadk3198 (2024)
DOI: 10.1126/sciadv.adk3198

The PDF file includes:

Figs. S1 to S11
Table S1
Legends for movies S1 to S3

Other Supplementary Material for this manuscript includes the following:

Movies S1 to S3

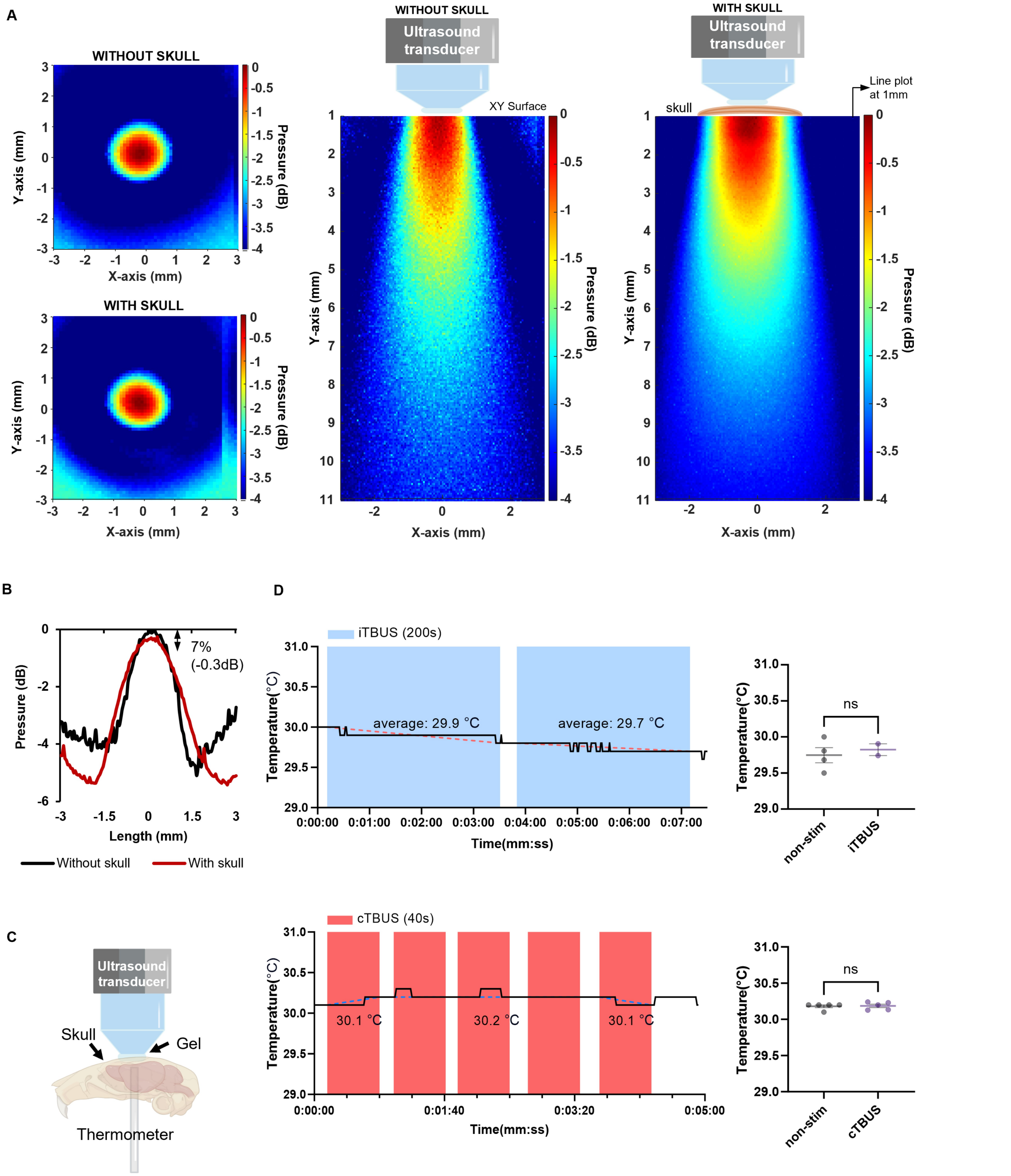

Fig. S1. Ultrasound Beam Profile and Temperature Change Measurement.
(A) Ultrasound pressure output is measured using a hydrophone in the water-based setup. The beam profile is presented in dB values on a Logscale to account for variations in the device's amplification values. (**B**) Ultrasound penetration through the skull leads to approximately a 7% decrease in pressure, corresponding to a reduction of 0.3 dB. (**C**) Schematic illustration of experiment. (**D**) Neither iTBUS nor cTBUS yields substantial heat change before and after stimulation.

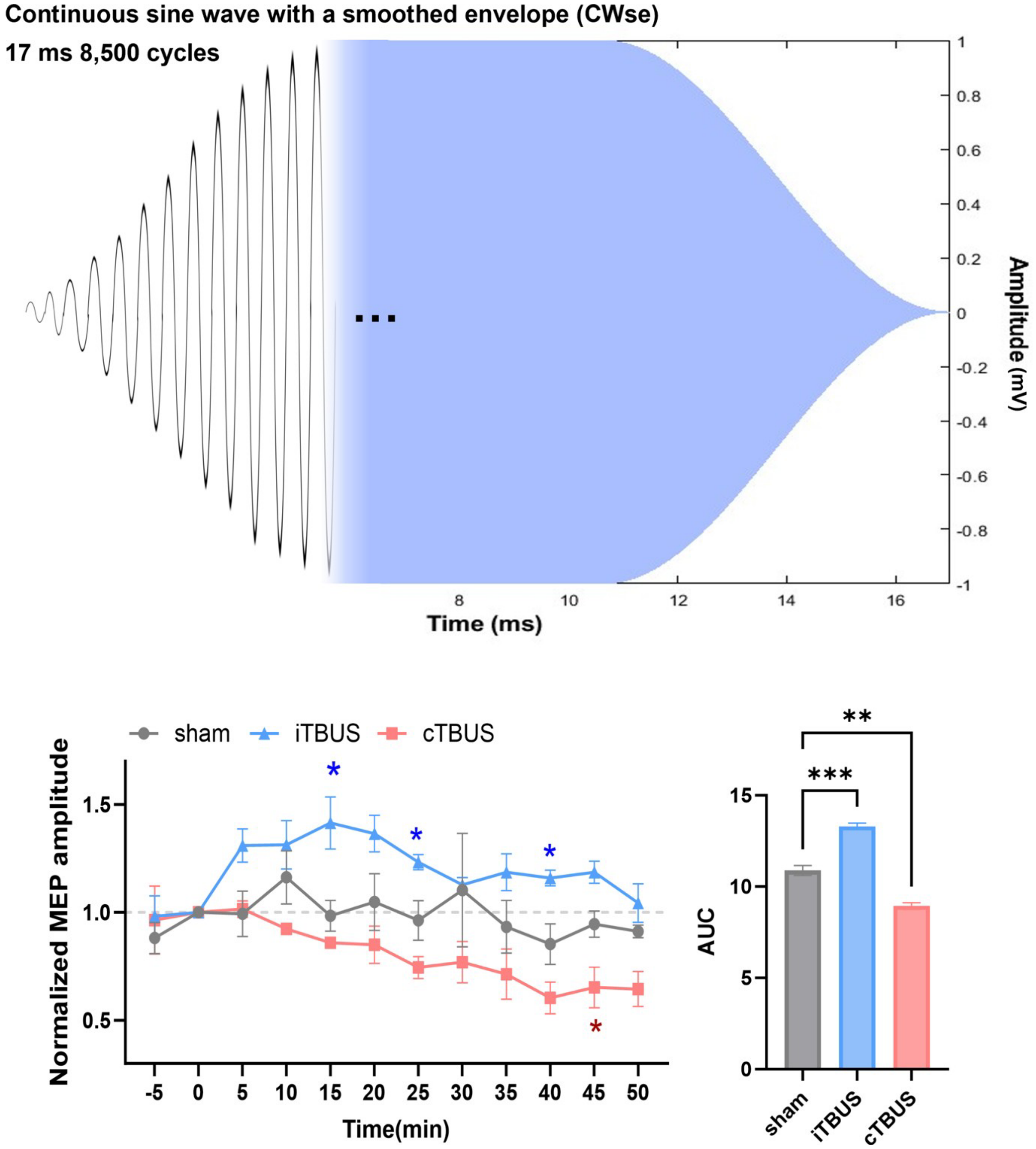

Fig. S2. Comparative Analysis of LTP- and LTD-Like Plasticity Induced by Smoothed Envelope Pulse Protocols. Pulse protocol generated using the smoothed envelope. Smoothed envelope pulse protocol resulted in the observation of LTP-like plasticity of MEP and LTD-like plasticity of MEP to a similar extent to iTBUS and cTBUS. AUC of normalized MEP amplitude was analyzed using an Ordinary one-way ANOVA, multiple comparison test, *** p = 0.0001, ns.

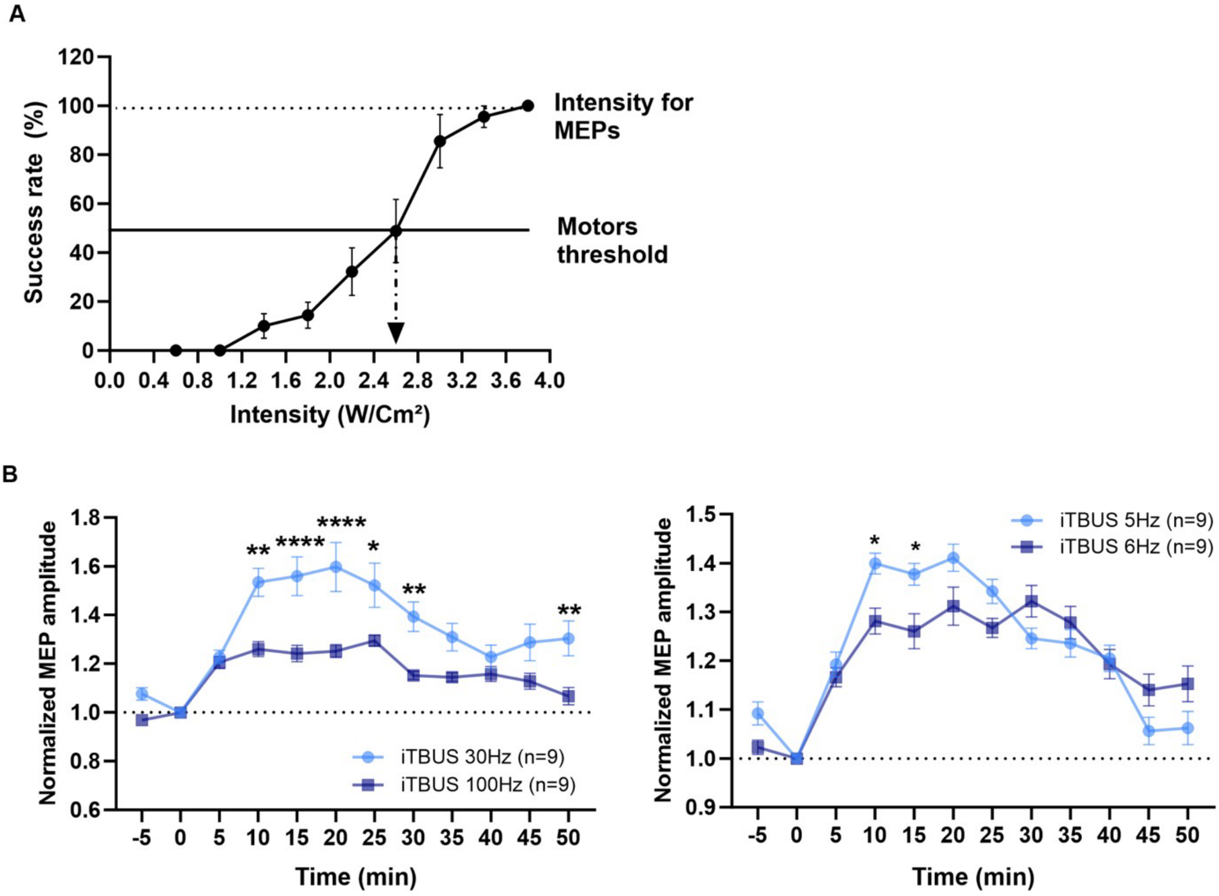

Fig. S3. Optimization of gamma and theta frequencies in the TBUS protocol. (**A**) A motor threshold was determined using a 5 ms ultrasound pulse in 8 animals to establish MEP and TBUS intensities. (**B**) After-effects of 100-Hz high gamma burst and 30-Hz low gamma, both coupled with 5-Hz theta, were compared in iTBUS (n=9). The 30-Hz group showed a prominent MEP increase up to 35 min, while the 100-Hz group showed an increase only within the first 5 min. Qualitative analysis and consistency led to the selection of 5-Hz theta frequency for the main experiments, as it exhibited better after-effect durations of up to 35 min. Data were presented as mean ± SED. *P* values were determined by Two-way ANOVA, *p<0.05, **p<0.01, ***p<0.001, ****p<0.0001.

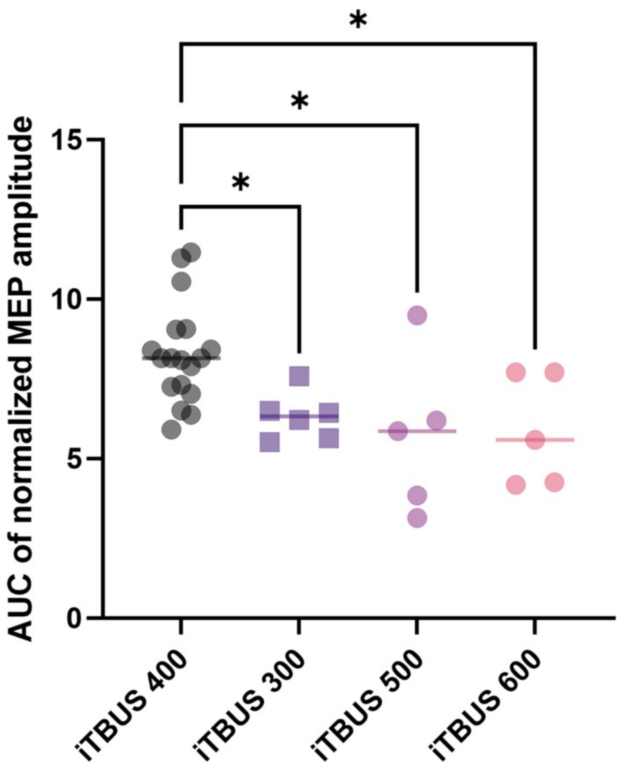

Fig. S4. Comparison of iTBUS effects using 4 pulses (iTBUS 400), 3 pulses (iTBUS 300), 5 pulses (iTBUS 500) and 6 pulses (iTBUS 600). Area under curve of MEP for iTBUS using 4 pulses (iTBUS 400, n = 18), 3 pulses (iTBUS 300, n = 6), 5 pulses (iTBUS 500, n = 5) and 6 pulses (iTBUS 600, n= 5). iTBUS 300, 500, and 600 effects were substantially lower than those from iTBUS 400, confirming the necessity of 4 pulses. P values were determined by Ordinary One-way ANOVA, multiple comparisons test, * p>0.01.

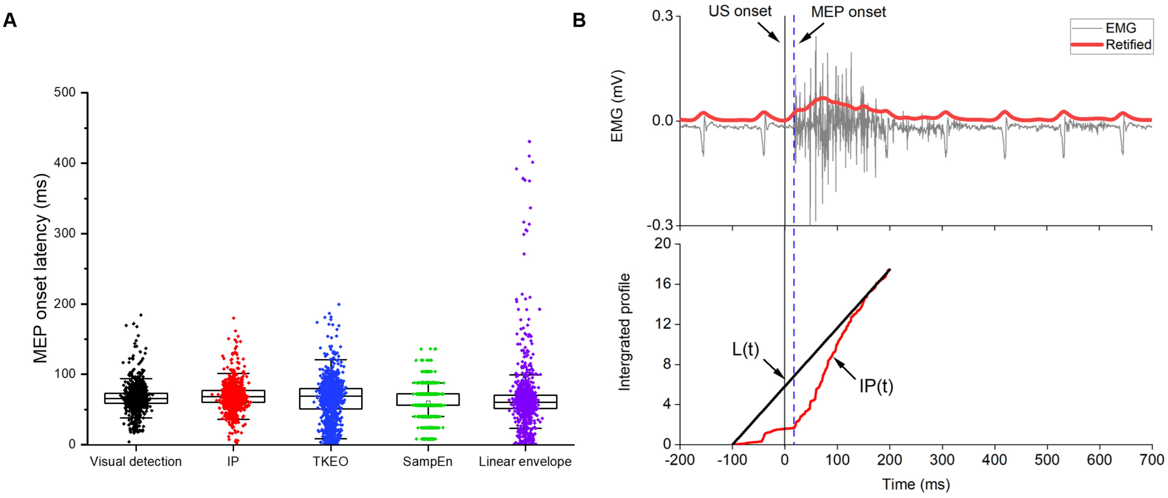

Fig. S5.MEP onset latency was investigated with 4 different methods: Integrated Profile, Teager-Kaiser Energy Operator (TKEO), Sample Entropy (SampEn), and Linear envelope. (**A**) In order to evaluate the performance of 4 different MEP onset detection methods, 1,200 MEP signals with varying levels of noise from 6 animals (200 MEPs in each animal) were visually inspected, and correct onset times were marked manually (visual detection method, black dots). The integrated profile method (IP, red dots) (*42*) utilized 2 lines: $\mathrm{IP}\,(t) = \sum_{\mathrm{i\#\$}}^{t} |x(i)|$ and $\mathrm{L}\,(t) = IP(M) \times \frac{t}{M}$, where IP (t) represents a continuous integration of all the rectified raw samples, x(i). The second line, L(t), indicates a reference line representing the integral of a signal equally distributed over the entire signal length (M). Then, using the following equation, D(t) = |IP(t) – L(t)|, MEP onset latency was defined as the point where D(t) reached its maximum value. Hence, the IP method detects the starting point of disproportionate slope increase for IP (t) (see Fig 1b for a graphical illustration of this method). The Teager-Kaiser Energy Operator method (TKEO, blue dots) (*47*) considered both amplitude and frequency information from an MEP signal in determining MEP onset latency. The following equation, Ψd [x(n)] = x2 (n) – x(n + 1)x(n – 1), where x is MEP signal and n is the sample number, was used for 10 – 500-Hz bandpass filtered and rectified MEP signals. Thus, TKEO output is proportional to the product of amplitude and frequency. The conventional threshold method of 3 sd was then applied to determine the onset latency for TKEO. Sample entropy (SamEn, green dots)(*48*) was calculated using a time window of 32 ms moving every 16 ms (hence 16 ms overlap). In each time window, sample entropy was calculated. A threshold of 0.5 was used to determine onset latency. Finally, the conventional linear envelope method used a threshold of 3 sd following 10-500 band-pass filtering, rectification, and smoothing using a zero-phase lag 2nd-order Butterworth filter with a cut-off frequency of 20-Hz. These different analyses with the same data revealed that SamEn data showed notably earlier onset latency than visual detection data. TKEO and linear envelope data exhibited higher variability compared with visual detection data. Hence, the IP method closely replicated our expert manual scoring. (**B**) MEP amplitude and onset latency analyses. Raw EMG signals were band-pass filtered (10-500-Hz), rectified, and smoothed (20-Hz). The peak amplitude of the resulting linear envelope represents motor cortex excitability. For MEP onset latency, the integrated profile was used to capture the starting point of sharp slope increase.

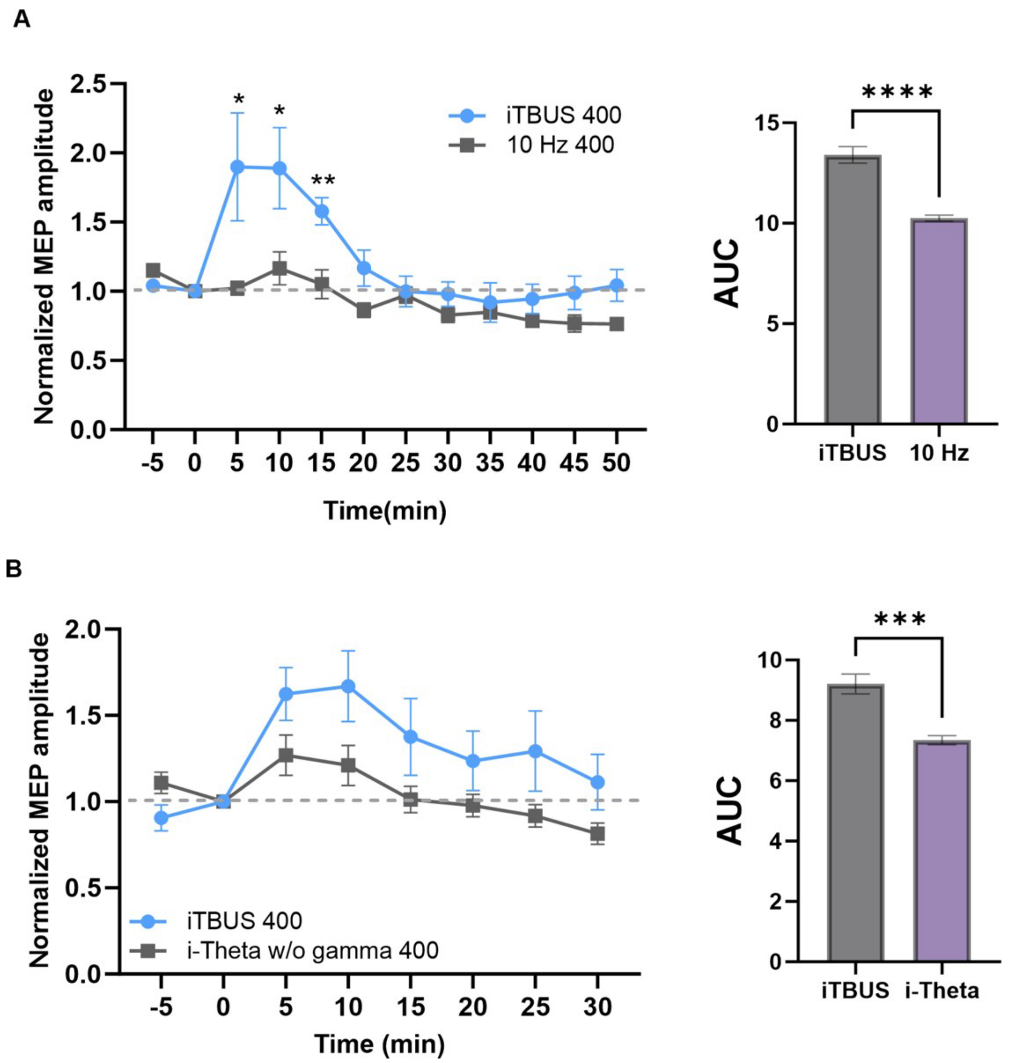

Fig. S6. Comparison between iTBUS and 10-Hz stimulation conditions and analysis of the effect of intermittent TBUS with and without gamma bursts. (**A**) Effects of iTBUS and 10-Hz stimulation (n=6 in each group). MEPs showed facilitation following iTBUS 400 pulses lasting for 20 minutes compared with baseline, whereas MEPs following 10-Hz stimulation were unchanged. *P* values were determined by Unpaired t-test per each time point, Two-tailed, *p<0.05, **p<0.005, ****p<0.0001. (**B**) Intermittent stimulation at 5-Hz with and without bursts, n=6 in each group (iTBUS vs. i-Theta). iTBUS was superior to i-Theta stimulation. Compared with the baseline, MEPs during the first 5 minutes following i-Theta were increased at a trend level, whereas MEPs showed facilitation following iTBUS 400 pulses. *P* values were determined by Unpaired t-test per each time point, Two-tailed, *p<0.05, **p<0.005, ****p<0.0001.

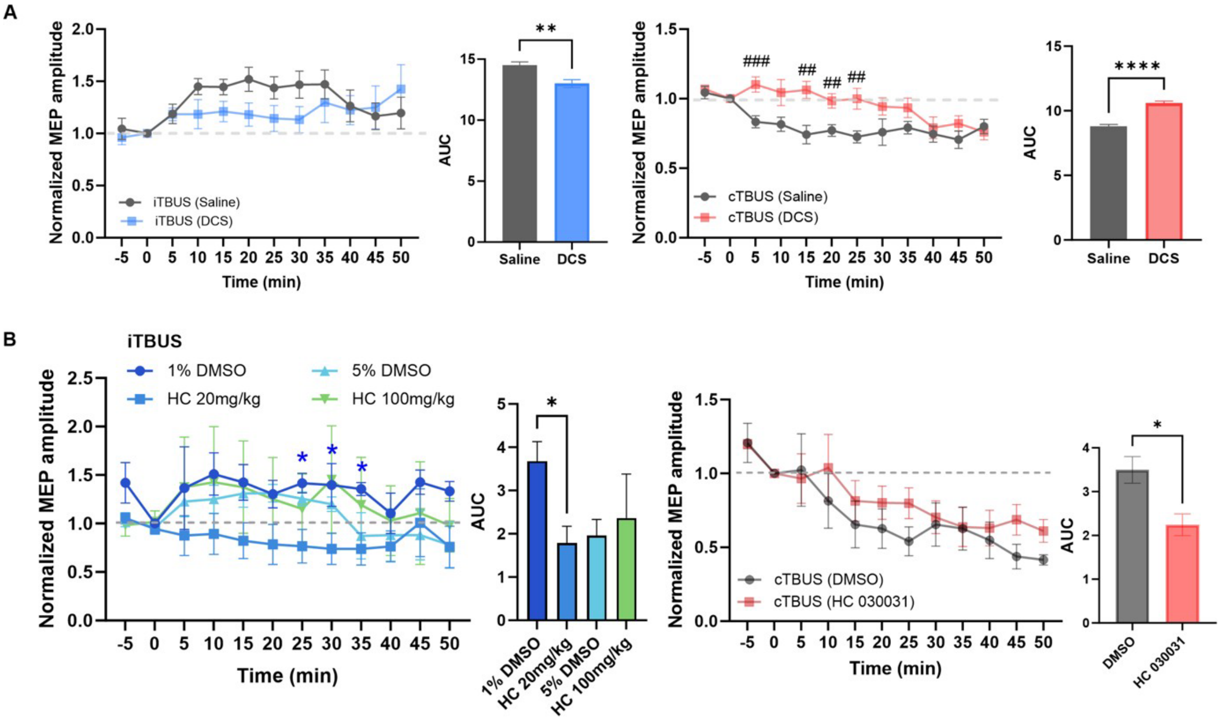

Fig. S7. Impact of NMDAR agonist and TrpA1 antagonist on iTBUS and cTBUS-induced plasticity in MEP amplitude. (**A**) Group data (n=6 in each group) of MEP amplitude for iTBUS (top) and cTBUS (bottom) with low-dose D-cycloserine (DCS), NMDAR agonist, treatment. In AUC of iTBUS, Data were presented as mean ± SEM. *P* values were determined by Unpaired t-test, Two-tailed, ** p=0.0042. In MEP amplitude of cTBUS, *P* values were determined by Unpaired t-test, two-tailed, ## p<0.005, ### p<0.001. In AUC of cTBUS, P values were determined by Unpaired t-test, Two-tailed, **** p<0.0001. (B) Group data of MEP amplitude for iTBUS (left) and cTBUS (right) with TrpA1 antagonist HC-030031 (i.p. 20 or 100 mg/kg). In iTBUS, 1% DMSO of n=4, HC 20 mg/kg of n=7, 5% DMSO of n=4 and HC 100 mg/kg of n=4. In cTBUS, DMSO is n=4, and HC-030031 is n=5. In MEP amplitude of iTBUS, *P* values were determined by unpaired t-test, two-tailed at each time point. * p<0.05. Both groups' AUCs and *P* values were determined by unpaired t-test, two-tailed * p=0.0133.

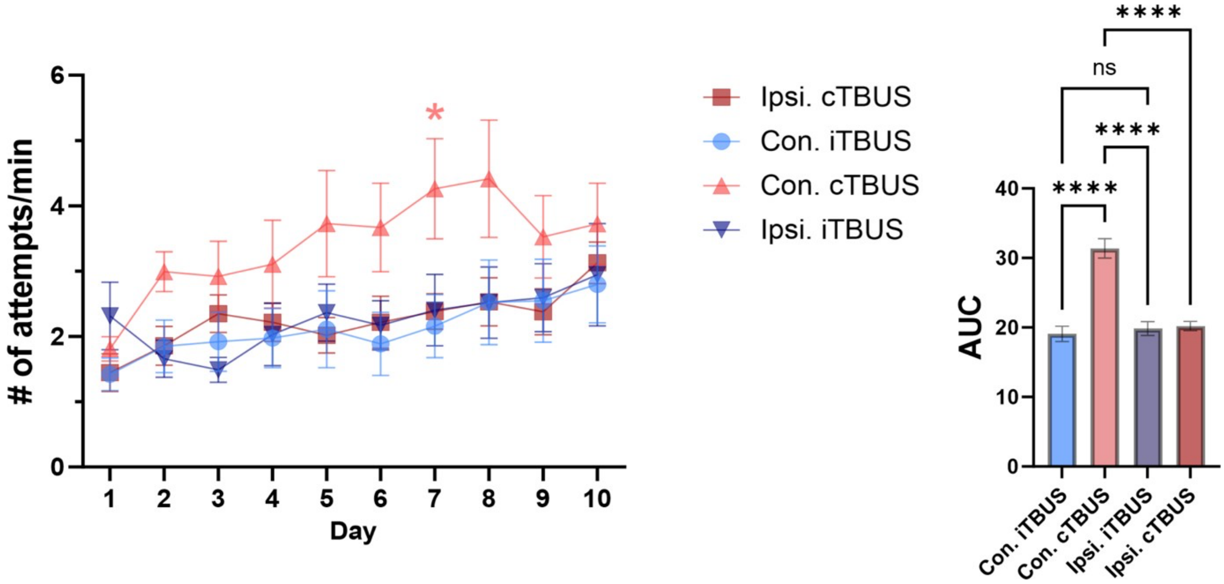

Fig. S8. Increased number of attempts during new motor skill learning following cTBUS. The cTBUS group exhibited more attempts in the contralateral conditions than the intermittent TBUS group, suggesting a compensatory mechanism for motor skill impairment. In the number of attempts/min graph, *P* values were determined by Unpaired t-test, two-tailed, each day point * p = 0.0342. AUC of attempts/min was analyzed using an Unpaired t-test, two-tailed, **** p < 0.0001, ns.

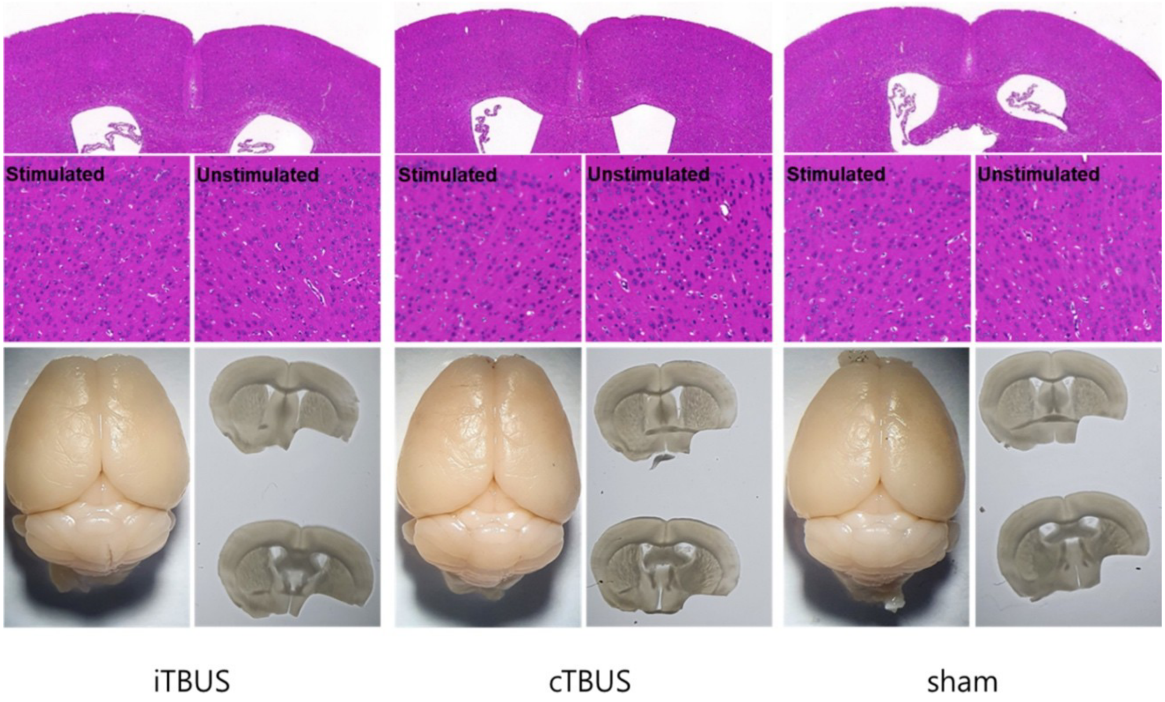

Fig. S9. The safety of TBUS was examined with hematoxylin and eosin (H&E) staining (upper and middle panels) and Evans blue dye (lower panels).
H&E staining was used to detect possible hemorrhaging or tissue damage after applying 800 pulses for iTBUS, cTBUS, and sham for 10 days (2 animals in each group). We did not find any evidence for tissue damage in any of the animals. This was consistent with the current literature on the safety of ultrasound stimulation, which states that the level of ultrasound intensity we used (0.29 MPa) was not associated with any safety concerns. Another 2 animals in each group underwent tail vein injection of Evans blue dye (EB) to test the presence of blood-brain barrier (BBB) disruption (lower panel). As presented, TBUS did not induce any observable alterations in EB distribution.

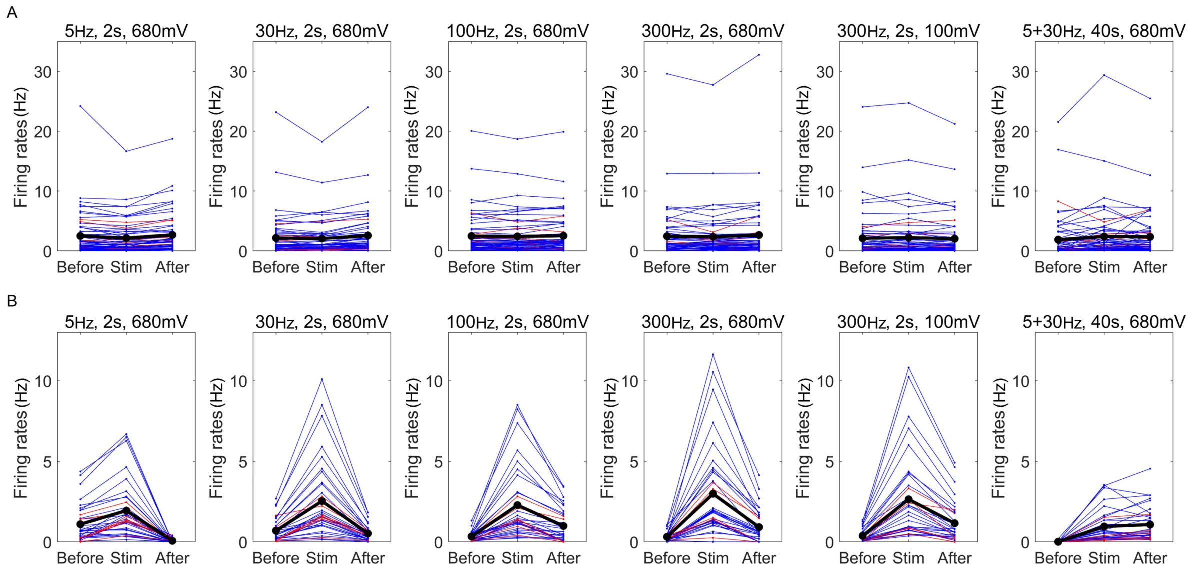

Fig. S10. Result of extracellular electrophysiology with ultrasound stimulation. Single units in the motor cortex (M1) were recorded with six different stimulation protocols under. (**A**) shallow anesthesia (1% at 1.0 liter/min) and (**B**) deep anesthesia condition (2% at 1.0 liter/min). Each colored line represents a single unit (red: narrow-spiking unit-Fast Spiking Unit (FSU), blue: broad-spiking unit-Regular Spiking Unit (RSU)). The black, thick line indicates the mean firing rates across single units. 680 mVrms corresponds to 2.621 W/cm^2, and 100 mVrms corresponds to 0.267 W/cm^2 (*61, 62*).

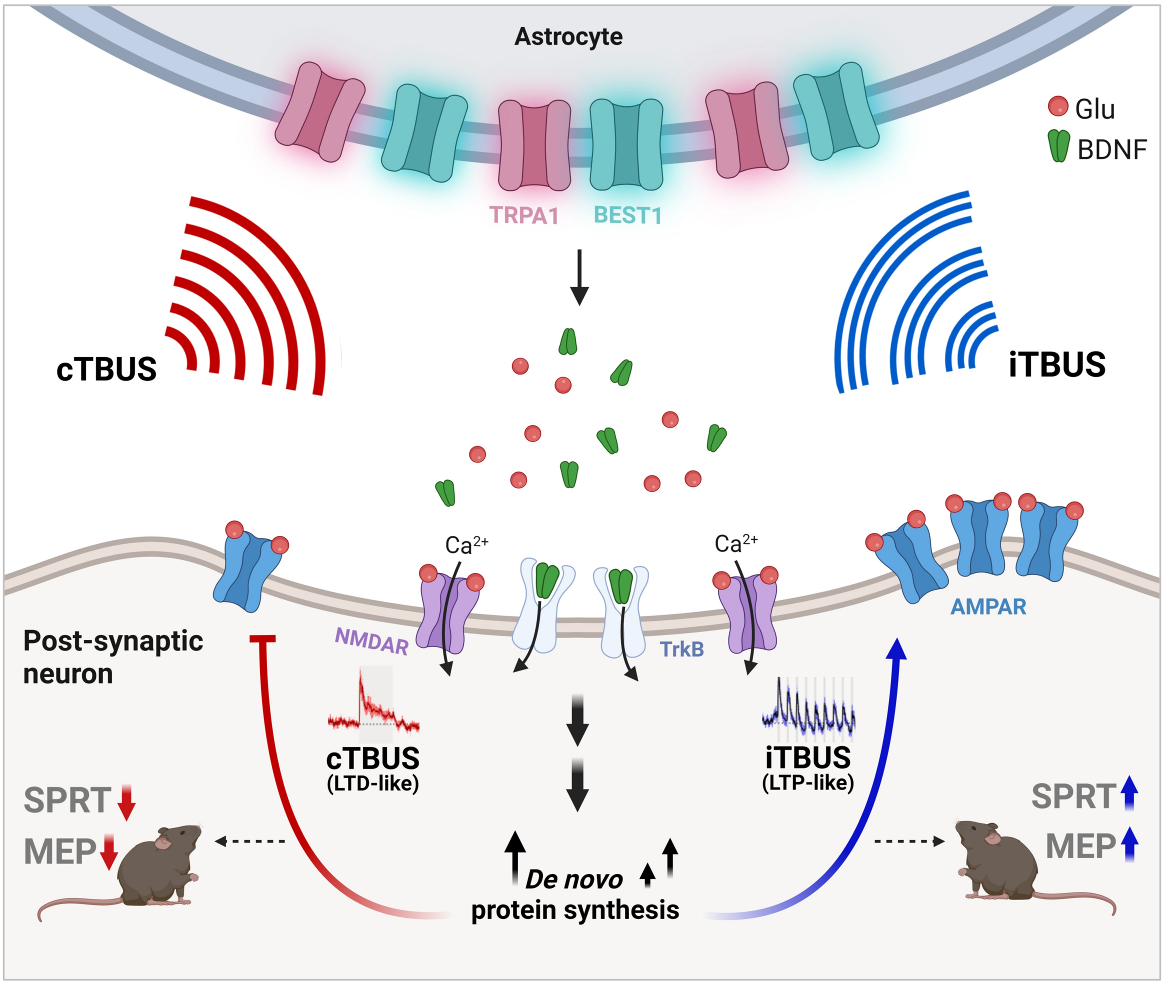

Fig. S11. Summary graphic abstract. There are two variations of TBUS—intermittent and continuous. Intermittent TBUS leads to long-term potentiation, while continuous TBUS induces depression-like plasticity, as seen through changes in motor-evoked potentials. These outcomes rely on molecular pathways linked to long-term plasticity, such as NMDA receptor and BDNF-TrkB activation, alongside de novo protein synthesis. Notably, Best1 and TRPA1 play crucial roles in sustaining these effects. Additionally, pre-training with TBUS enhances the acquisition of new motor skills.

		Contralateral		Ipsilateral	
		iTBUS (n = 9)	cTBUS (n = 9)	iTBUS (n = 9)	cTBUS (n = 9)
Shaping speed	Fast	3	4	3	4
	Intermediate	4	4	4	4
	Slow	2	1	2	1
Forelimb dominance	Left	5	5	5	3
	Right	4	4	4	6

Table S1. Individual variations of performance during shaping sessions. This table represents mice's dominant preference and inherent learning ability in the SPRT task. "Fast" refers to mice that determined their forelimb dominance within 1-2 days of shaping, "Intermediate" refers to mice within 3-5 days, and "Slow" refers to mice within 6-7 days. Due to the differences in learning abilities associated with the speed of the paw preference determination, the mice were evenly distributed among the groups. Similarly, the ratio of right-handed and left-handed mice was evenly distributed among the groups.

Video

S1. This video displays SPRT in the sham control group. Out of a total of 30 trials, it reveals 10 trials depicting success and failure. The mice did not receive ultrasound stimulation, but anesthesia and head shaving were conducted for comparison purposes. this video features a representative mouse in the group on the 6th day of training.

S2. This video displays SPRT in the iTBUS group. Out of a total of 30 trials, it reveals 10 trials depicting success and failure. The ultrasound was administered 30 minutes prior to the start of the experiment, and this video features a representative mouse in the group on the 6th day of training.

S3. This video displays SPRT in the cTBUS group. Out of a total of 30 trials, it reveals 10 trials depicting success and failure. The ultrasound was administered 30 minutes prior to the start of the experiment, and this video features a representative mouse in the group on the 6th day of training.
